# Supplementary material for: A DAP5/eIF3d alternate mRNA translation mechanism promotes differentiation and immune suppression by human regulatory T cells
Source: Nat Commun. 2021 Nov 30;12:6979. doi: 10.1038/s41467-021-27087-w (PMC8632918; doi:10.1038/s41467-021-27087-w)
Supplement: Supplementary file 1 — Supplementary Information [file 41467_2021_27087_MOESM1_ESM.pdf]

A DAP5/eIF3d alternate mRNA translation mechanism promotes differentiation and immune suppression function of human regulatory T cells

Viviana Volta[1,2,7], Sandra Pérez-Baos[1,7], Columba de la Parra[1,3], Olga Katsara[1], Amanda Ernlund[1,4], Sophie Dornbaum[1] and Robert J. Schneider[1,5,6]

[1] Department of Microbiology, NYU Grossman School of Medicine, New York, NY, 10016, USA. [2] Synthis LLC, 430 East 29th Street, Launch Labs, Alexandria Center for Life Sciences, New York, NY, 10016 USA. Department of Chemistry, Herbert H. Lehman College and The Graduate Center, Biochemistry Ph.D. [3]Program, City University of New York, New York, NY 10016 USA. [4] Johns Hopkins Applied Physics Lab, 11000 Johns Hopkins Road, Laurel, MD 20723 USA. [5] Perlmutter Cancer Center, NYU Grossman School of Medicine, New York, NY, 10016, USA. [6] Colton Center for Autoimmunity, NYU Grossman School of Medicine, New York, NY, 10016, USA. [7] These authors contributed equally: Viviana Volta, Sandra Pérez-Baos

Corresponding author: Dr. Robert J. Schneider, NYU School of Medicine, Alexandria Center for Life Science, 450 East 29th Street, New York, NY 10016; Tel: 212-263-6006; Fax: 46-501-4541; email: robert.schneider@nyumc.org

Supplementary Figures 1-8

Supplementary Table 1

Supplementary Table 2

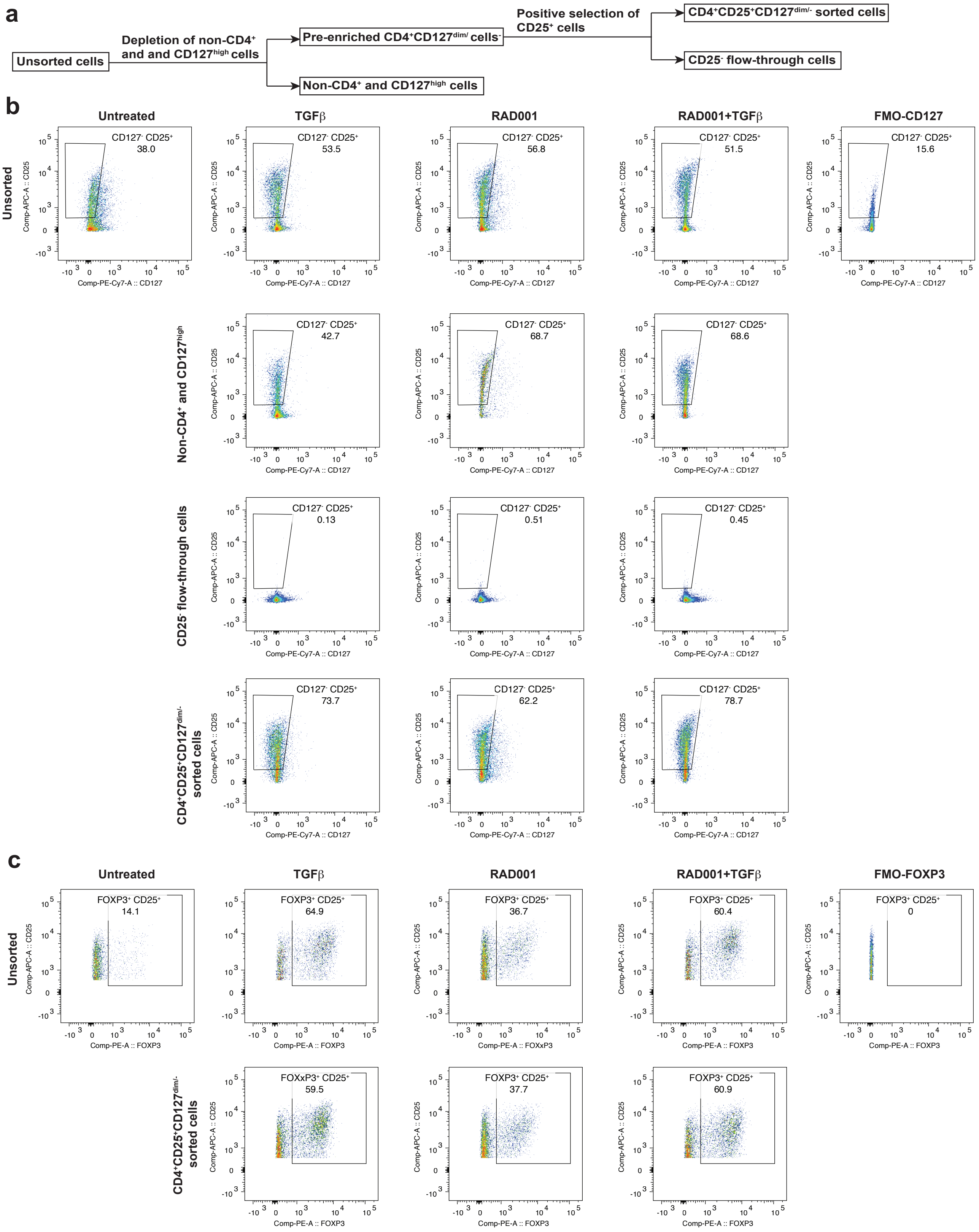

Supplementary Fig. 1. Analysis of Treg cell markers for T cell populations generated during Treg cell differentiation and enrichment. a Scheme of naïve CD4+ T cell isolation strategy using magnetic sorting. Both the negative and positive populations were collected at every step: CD4+CD127low and the CD4-CD127high cells for the pre-enrichment step, and CD25+ and CD25- cells for the CD25 selection. **b, c** Cells were generated, isolated and labeled as described in Fig. 2 legend. Flow cytometry analysis of all the collected populations is shown, including the unsorted cells, the intermediate steps and the non-Treg cells, for each treatment. Gating for markers CD25/CD127 as in **b** and FOXP3 as in **c**. (**b, c**) Representative of >70 independent donor T cell isolations.

Supplementary Fig. 2 (page 1 of 3)

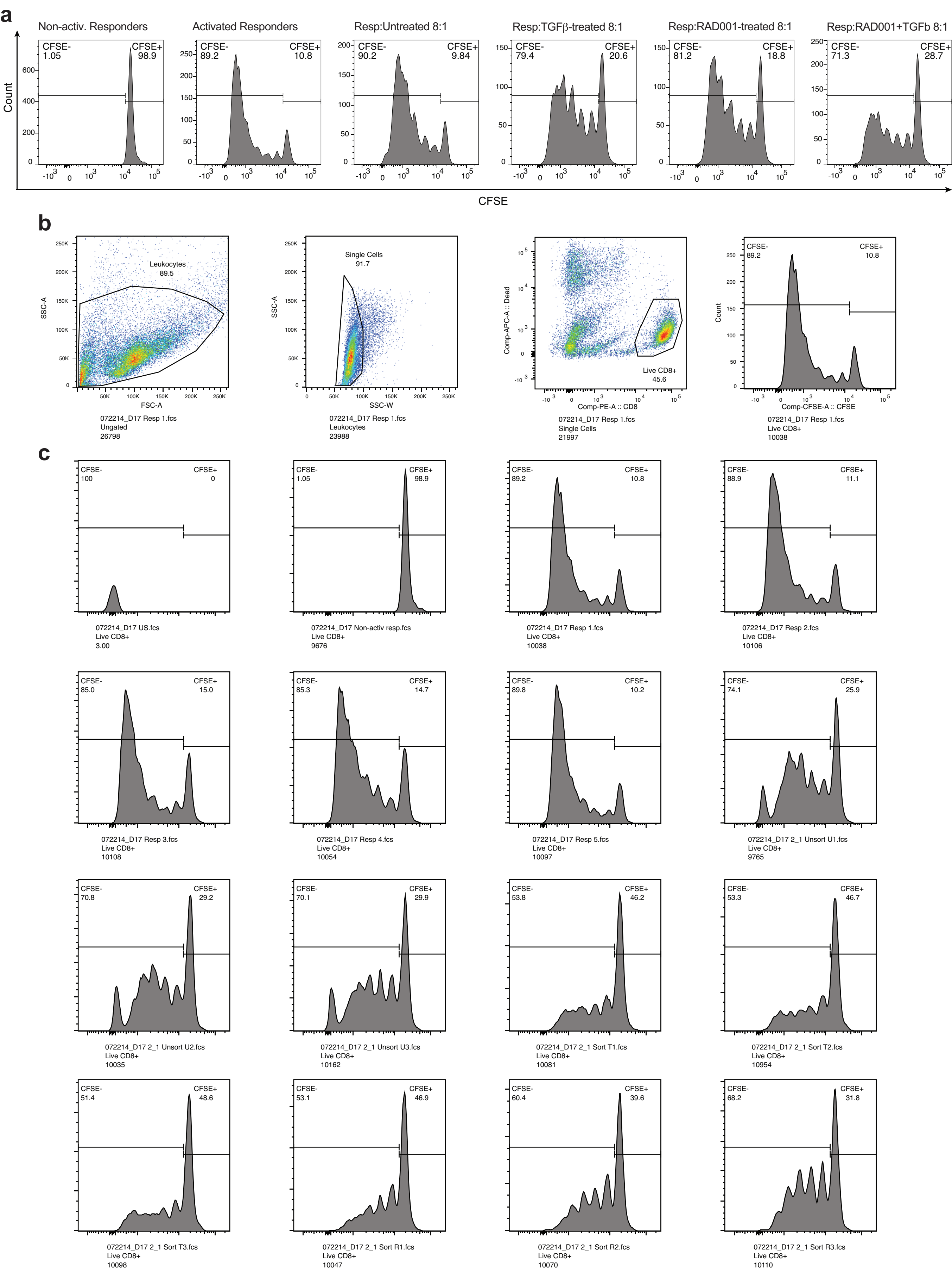

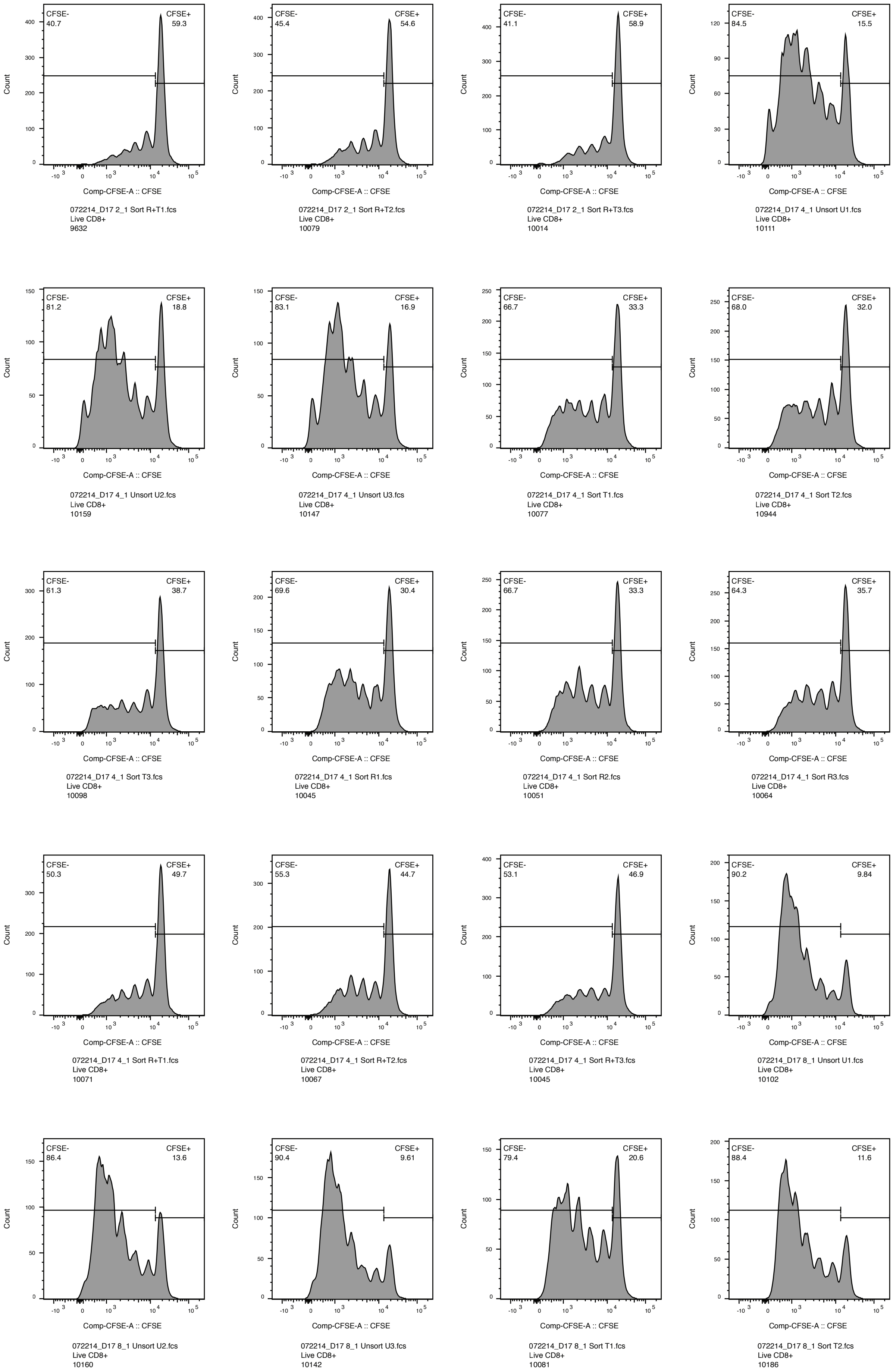
CFSE- 40.7 CFSE+ 59.3
Count
Comp-CFSE-A :: CFSE
072214_D17 2_1 Sort R+T1.fcs
Live CD8+
9632
CFSE- 45.4 CFSE+ 54.6
072214_D17 2_1 Sort R+T2.fcs
Live CD8+
10079
CFSE- 41.1 CFSE+ 58.9
072214_D17 2_1 Sort R+T3.fcs
Live CD8+
10014
CFSE- 84.5 CFSE+ 15.5
072214_D17 4_1 Unsort U1.fcs
Live CD8+
10111
CFSE- 81.2 CFSE+ 18.8
072214_D17 4_1 Unsort U2.fcs
Live CD8+
10159
CFSE- 83.1 CFSE+ 16.9
072214_D17 4_1 Unsort U3.fcs
Live CD8+
10147
CFSE- 66.7 CFSE+ 33.3
072214_D17 4_1 Sort T1.fcs
Live CD8+
10077
CFSE- 68.0 CFSE+ 32.0
072214_D17 4_1 Sort T2.fcs
Live CD8+
10944
CFSE- 61.3 CFSE+ 38.7
072214_D17 4_1 Sort T3.fcs
Live CD8+
10098
CFSE- 69.6 CFSE+ 30.4
072214_D17 4_1 Sort R1.fcs
Live CD8+
10045
CFSE- 66.7 CFSE+ 33.3
072214_D17 4_1 Sort R2.fcs
Live CD8+
10051
CFSE- 64.3 CFSE+ 35.7
072214_D17 4_1 Sort R3.fcs
Live CD8+
10064
CFSE- 50.3 CFSE+ 49.7
072214_D17 4_1 Sort R+T1.fcs
Live CD8+
10071
CFSE- 55.3 CFSE+ 44.7
072214_D17 4_1 Sort R+T2.fcs
Live CD8+
10067
CFSE- 53.1 CFSE+ 46.9
072214_D17 4_1 Sort R+T3.fcs
Live CD8+
10045
CFSE- 90.2 CFSE+ 9.84
072214_D17 8_1 Unsort U1.fcs
Live CD8+
10102
CFSE- 86.4 CFSE+ 13.6
072214_D17 8_1 Unsort U2.fcs
Live CD8+
10160
CFSE- 90.4 CFSE+ 9.61
072214_D17 8_1 Unsort U3.fcs
Live CD8+
10142
CFSE- 79.4 CFSE+ 20.6
072214_D17 8_1 Sort T1.fcs
Live CD8+
10081
CFSE- 88.4 CFSE+ 11.6
072214_D17 8_1 Sort T2.fcs
Live CD8+
10186

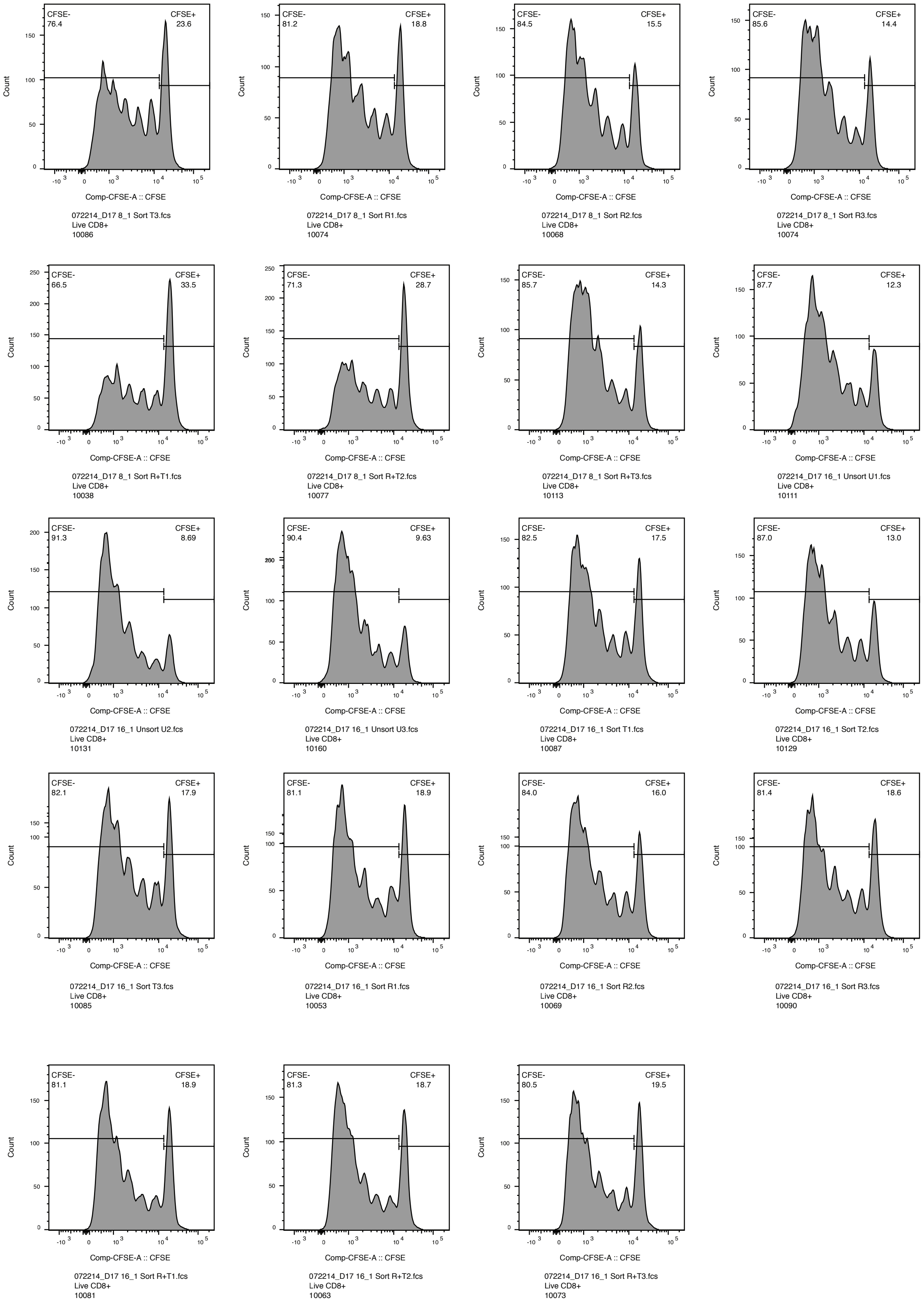

Supplementary Figure 2. Immune suppression assay results. a Suppression assays representative of 3 studies were performed as described in Fig. 3 legend and Methods. CFSE-labeled responders were stained for live CD8+ cells and visualized by flow cytometry. **b** Gating strategy for analysis of CD8+ T cells used for responder assays. **c** CSFE profiles for individual samples: inactivated responders (plated without anti-CD3 and anti-CD28), average of five different donor activated responders (cultured alone), and all the responders co-cultured with cells from the four treatments (untreated, TGFβ, RAD001, R+T) at ratios shown in Fig. 3.

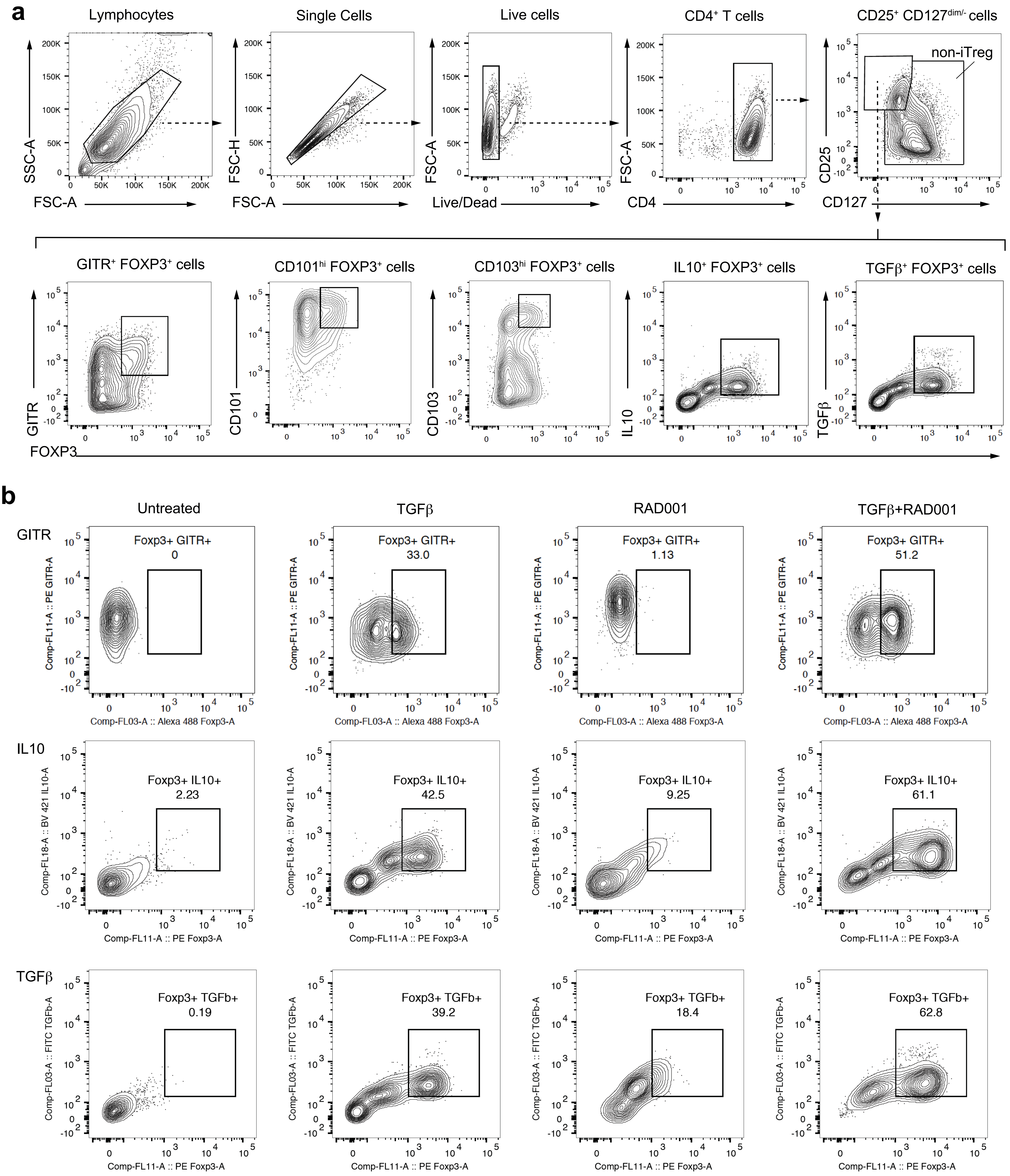

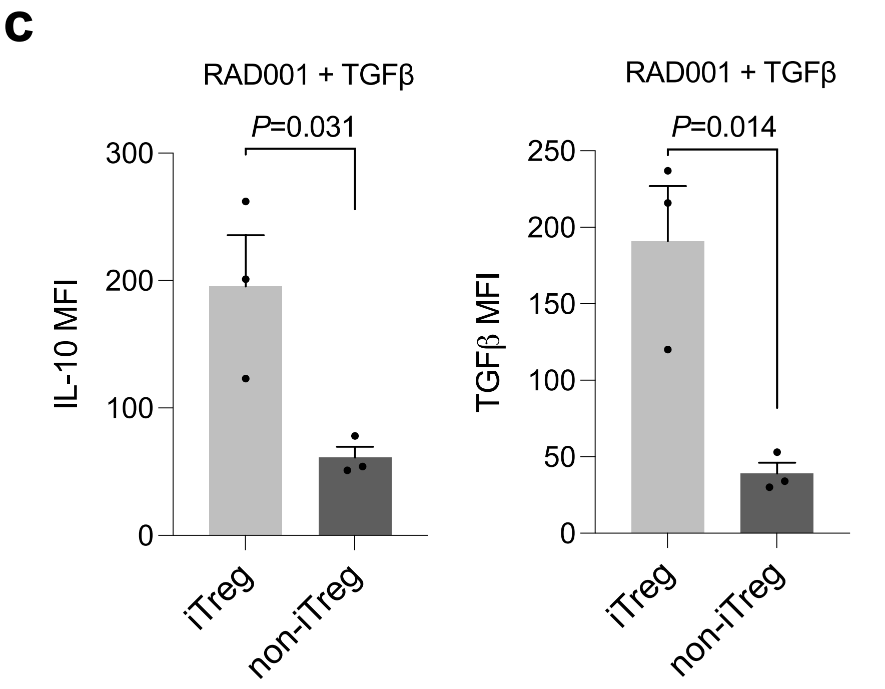

Supplementary Fig. 3. Representative flow cytometry analysis of Treg cell markers during Treg cell differentiation and enrichment in culture from naïve CD4+ T cells. **a** Gating strategy for analysis of Treg and non-Treg cell populations and expression levels of GITR, CD101, CD103, IL10 and TGFβ. Representative images shown from n=3 indepen-dent experiments. **b** Effect of treatment on Treg cell markers GITR, IL10 and TGFβ starting with naïve CD4+ T cells treated with TGFβ, RAD001, both or vehicle (untreated). Representative images shown. **c** Comparison of Treg and non-Treg cell populations for expression of IL-10 and TGFβ after induction of Treg cell differentiation. Data represent mean and SEM of 3 independent experiments. Statistical analysis by two-way ANOVA test with Dunnett post-ANOVA test determination.

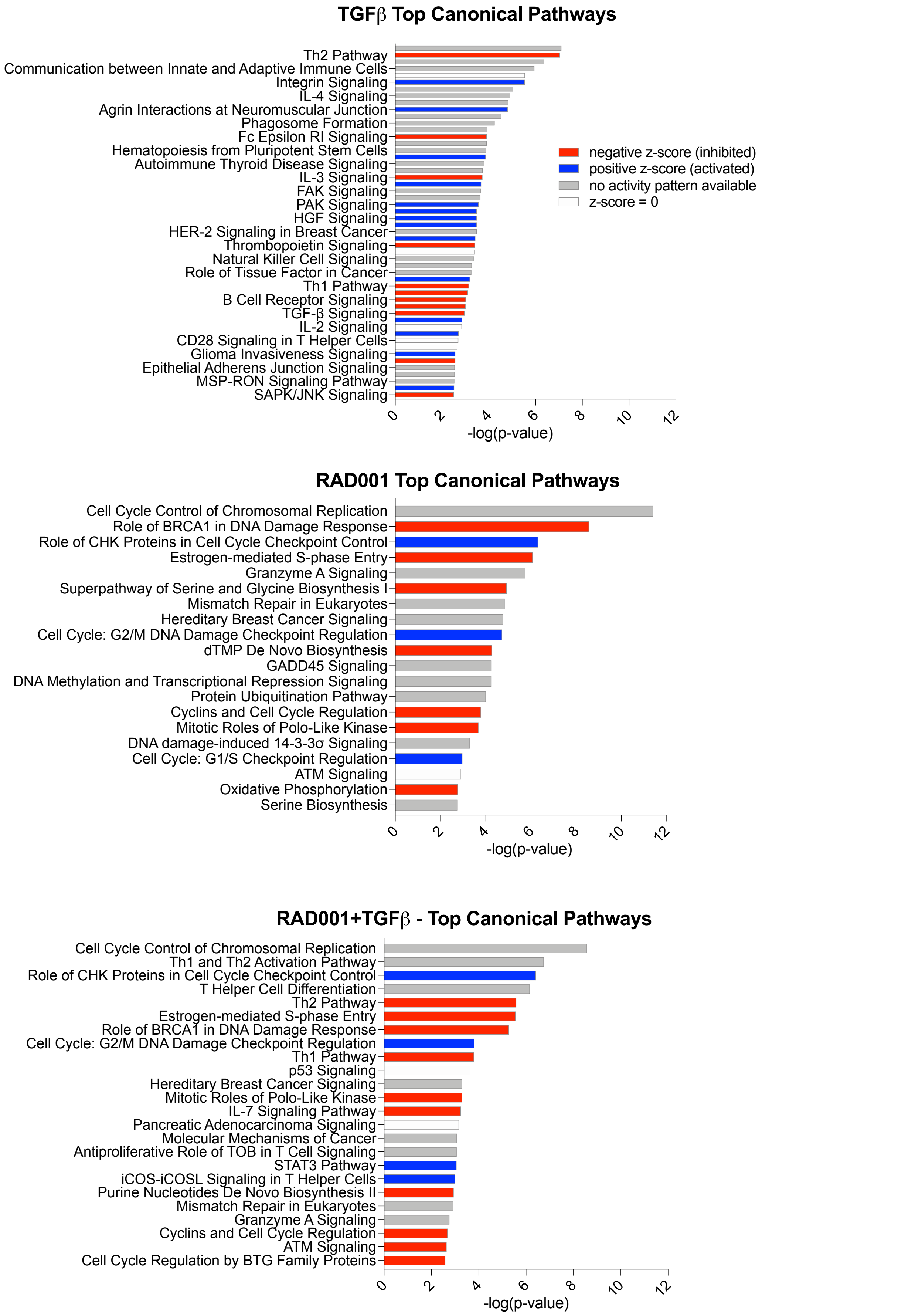

Supplementary Fig. 4. Analysis of signaling pathways across the different treatment groups using genome-wide translatome data. Highly significant pathways [-log(p-value) ≥ 2.5 from Ingenuity Pathway Analysis software calculations] are included. Data analysis involved use of GCCN and SST transformation algorithms, RMA background correction, limma R package statistics, linear regression models, Fisher's exact tests, Benjamin-Hochberg corrections for multiple analysis. Color code for the histograms refers to the z-score calculated by the software when possible, indicating the predicted activation state of each pathway.

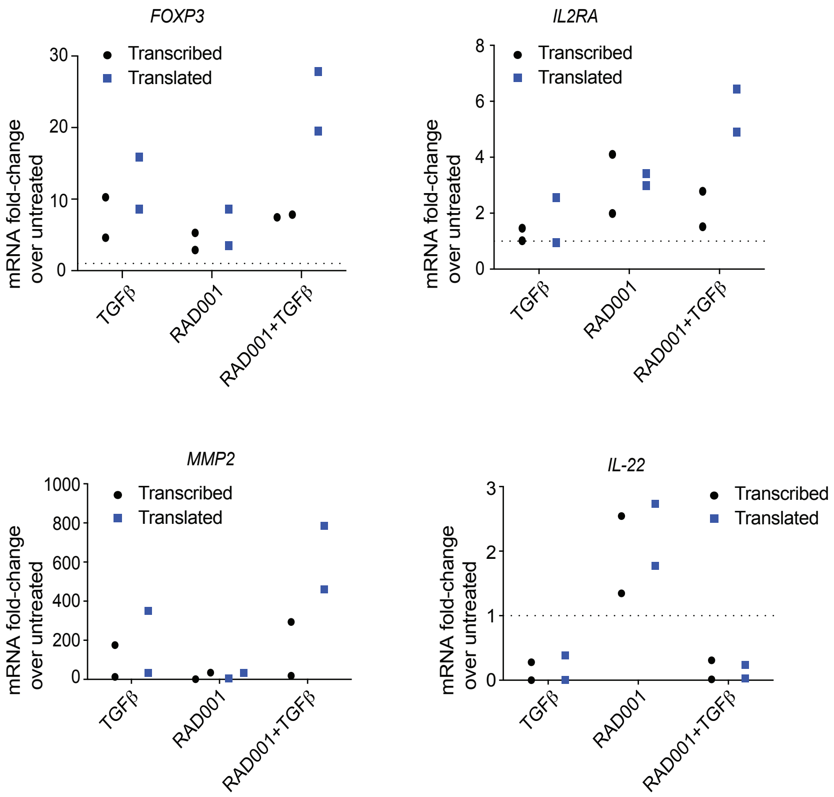

Supplementary Figure 5. Validation of genome-wide data obtained by measuring mRNA abundance (transcribed) and enrichment in polysomal fractions (translated) using qRT-PCR analysis. FOXP3, IL2RA, MMP2 trend to upregulation, and IL-22 trend to downregulation in TGFβ plus RAD001 treated $CD4^+$ T cells were confirmed with RNA samples used for genome-wide studies.Data represent 2 independent studies.

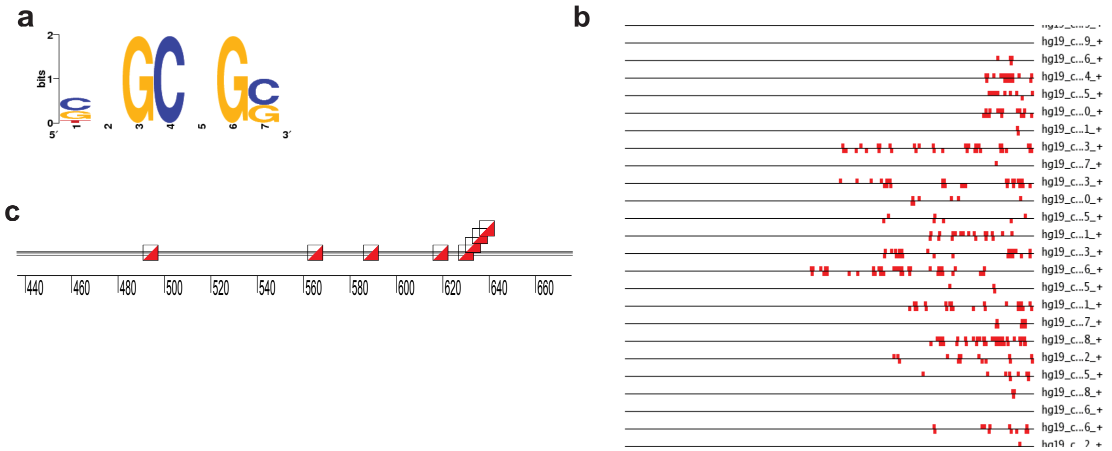

Supplementary Figure 6. Non-canonical Treg cell mRNAs are enriched in repeating GCxGCn 5'UTR sequences. a Sequence logo of motif found enriched in some 5'UTRs of non-canonical Treg cell mRNAs that were well translated during differentiation. Shown is the sequence cluster in red in the 5'UTR of the *PRICKLE* mRNA. The sequence logo represents the motif found by analyzing the 5'UTR of the genes represented in **a** via the tool SCOPE. **b** Examples of Treg cell non-canonical mRNAs harboring the highly repeated motif in their 5' UTR (each red square represents the motif). For each gene, all the variants were included in the analysis. **c** Schematic representation of the GC-rich region pattern in the *PRICKLE* 5'UTR.

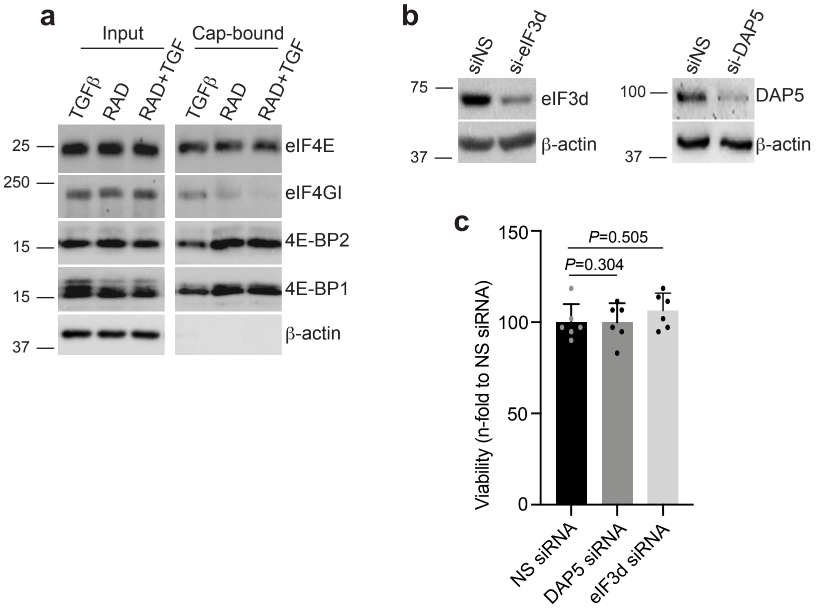

Supplementary Fig. 7. Effects of RAD001 + TGFβ on canonical eIF4E cap binding and effect of silencing DAP5 and eIF3d on viability of 293T cells. a Human naïve $CD4^+$ T lymphocytes were differentiated into Tregs with TGFβ and RAD001 as described in the legend to Fig. 2**a**. Equal protein amounts were subjected to cap-chromatography normalized to equal levels of eIF4E retention. Shown are representative immunoblots of 3 independent donors of input and cap-retained proteins, subjected to immunoblot analysis as indicated. **b** HEK293 cells were transfected with siRNAs to *eIF3d*, DAP5 (*eIF4G2*) or control non-silencing (Nsi). Cells were harvested at 72 h post-transfection, and equal amounts of protein lysates subjected to immunoblot analysis. Representative image of 3 studies. **c** Following 72 h of silencing, 293 cells were subjected to viability analysis by Trypan Blue exclusion assay. There is no statistical difference in percent viability with silencing of *eIF3d* or *eIF4G2* mRNAs over 72 h. Data represent mean and SEM of 3 indpendent experiments. Statistical analysis by two-way ANOVA test with Dunnett post-ANOVA test determination.

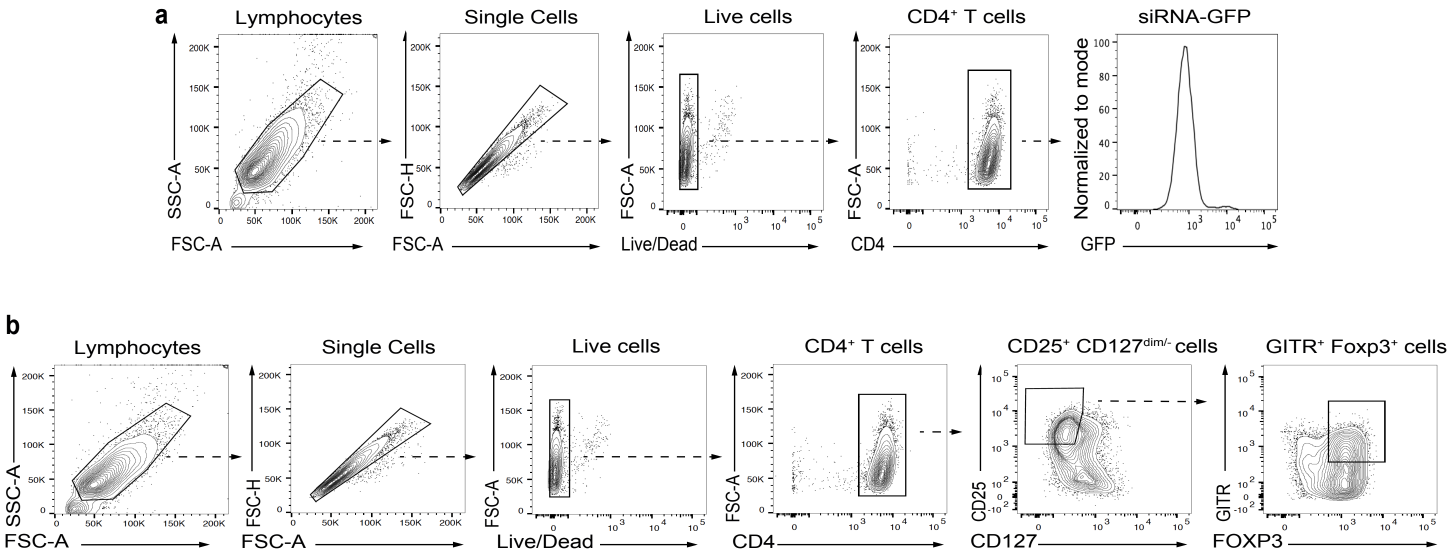

Supplementary Figure 8. Representative flow cytometry analysis of $CD4^+$ T cell Treg cell differentiation silenced for DAP5/ *eIF4G2* mRNA. **a** Gating strategy for analysis of $CD4^+$ T cells and ex vivo quantification of transfection by control or *eIF4G2* targeting siRNAs. Freshly isolated human naïve $CD4^+$ T cells were partially silenced for DAP5 expression by repeated treatment with a pool of Accell SMARTpool siRNAs targeting *eIF4G2* mRNA or a non-targeting siRNA pool in serum-free X-Vivo 15 media 3 d apart and stimulated with IL-2, TGFβ and RAD001. Representative images shown of 2 independent studies. A GFP non-targeting siRNA was used to verify T cell uptake of siRNAs. **b** Gating strategy used to analyze the effect of siRNA treatment on differentiation of Treg cells. Representative images shown of 2 independent studies.

Supplementary Table 1: Functions of non-canonical DAP5/eIF3d-dependent mRNAs

Symbol	Entrez Gene Name (ID)	Gene Function
PRICKLE1	Prickle Planar Cell Polarity Protein 1 (#144165)	Nuclear receptor that may be a negative regulator of the Wnt/beta-catenin signaling pathway. The encoded protein localizes to the nuclear membrane and has been implicated in the nuclear trafficking of the transcription repressors REST/NRSF and REST4. Mutations in this gene have been linked to progressive myoclonus epilepsy.
PTCHD1	Patched domain containing 1 (#139411)	Membrane protein with a patched domain. The encoded protein is similar to Drosophila proteins which act as receptors for the morphogen sonic hedgehog. Deletions in this gene, which is located on the X chromosome, are associated with intellectual disability and autism.
IRS2	Insulin receptor substrate 2 (#8660)	A cytoplasmic signaling molecule that mediates effects of insulin, insulin-like growth factor 1, and other cytokines by acting as a molecular adaptor between diverse receptor tyrosine kinases and downstream effectors. The product of this gene is phosphorylated by the insulin receptor tyrosine kinase upon receptor stimulation, as well as by an interleukin 4 receptor-associated kinase in response to IL4 treatment.
ATP1B1	ATPase, Na+/K+ transporting, beta 1 polypeptide (#481)	ATPase belonging to the family of Na+/K+ and H+/K+ ATPases beta chain proteins, and to the subfamily of Na+/K+ -ATPases. Na+/K+ -ATPase is an integral membrane protein responsible for establishing and maintaining the electrochemical gradients of Na and K ions across the plasma membrane. These gradients are essential for osmoregulation, for sodium-coupled transport of a variety of organic and inorganic molecules, and for electrical excitability of nerve and muscle.
SLC16A2	Solute carrier family 16, member 2 (thyroid hormone transporter) (#6567)	Integral membrane protein that functions as a transporter of thyroid hormone. The encoded protein facilitates the cellular importation of thyroxine (T4), triiodothyronine (T3), reverse triiodothyronine (rT3) and diidothyronine (T2). This gene is expressed in many tissues and likely plays an important role in the development of the central nervous system. Loss of function mutations in this gene are associated with psychomotor retardation in males while females exhibit no neurological defects and more moderate thyroid-deficient phenotypes. This gene is subject to X-chromosome inactivation.
SCML1	Sex comb on midleg-like 1 (Drosophila) (#6322)	Putative Polycomb group (PcG) protein. PcG proteins act by forming multiprotein complexes, which are required to maintain the transcriptionally repressive state of homeotic genes throughout development. May be involved in spermatogenesis during sexual maturation.
GJB6	Gap junction protein, beta 6, 30kDa (connexin-30) (#10804)	Gap junctions allow the transport of ions and metabolites between the cytoplasm of adjacent cells. They are formed by two hemichannels, made up of six connexin proteins assembled in groups. The specificity of the gap junction is determined by which connexin proteins comprise the hemichannel. This gene encodes one of the connexin proteins. Mutations in this gene have been found in some forms of deafness and in some families with hidrotic ectodermal dysplasia

Supplementary Table 2: Primers and oligonucleotides

**Oligonucleotides (5' to 3' shown)**		
	**Source**	**Cat. #**
FOXP3 forward primer 5'-CATGCGACCCCCTTTCAC-3'<br>reverse primer 5'-AGATCTCATTGAGTGTCCGCT-3'	This paper	N/A
IL2RA forward primer 5'-ACAGAGTACCAGGTAGCAG-3'<br>reverse primer 5'-TACTCTTCCTCTGTCTCCG-3'	This paper	N/A
MMP2 forward primer 5'-GTTGGCAGTGCAATACCTG-3'<br>reverse primer 5'-GGCAGTCCAAAGAACTTCTG-3'	This paper	N/A
IL-22 forward primer 5'-GGTTCCAGCCTTATATGCAG-3'<br>reverse primer 5'-CACCTTCAATATGACATGTGC-3'	This paper	N/A
GAPDH forward primer 5'-TTAAAAGCAGCCCTGGTGAC-3'<br>reverse primer 5'-CTCTGCTCCTCCTGTTCGAC-3'	This paper	N/A
PRICKLE construction primers:<br>forward primer 5'-CTTAAGAGCGGAGACGAGTTTTTC-3'<br>reverse primer 5'AGATATCGGTTTCTCAGTCACAGGACATC-3'	This paper	N/A
ITGB1 construction primers:<br>Forward primer 5'-<br>CATTCCGGTACTGAATTGATGAGCCAGCCCAGCCGCGT -3'<br>reverse primer 5'-<br>CATGGTGGCTAGAGCTTGATCTGCGCTTGCCCAGCCCG -3'	This paper	N/A
Renilla primers for qRT-PCR:<br>forward primer 5'-TAA CTG GTC CGC AGT GGT GGG-3'<br>reverse primer 5'-CAG CAT TTT CTG CAT GTT TTT CT-3'	This paper	N/A
