# Supplementary material for: A DAP5/eIF3d alternate mRNA translation mechanism promotes differentiation and immune suppression by human regulatory T cells
Source: Nat Commun. 2021 Nov 30;12:6979. doi: 10.1038/s41467-021-27087-w (PMC8632918; doi:10.1038/s41467-021-27087-w)
Supplement: Supplementary file 3 — Description of Additional Supplementary Files [file 41467_2021_27087_MOESM3_ESM.pdf]

Description of Additional Supplementary Files

File Name: Supplementary Data 1
Description: Table containing raw expression data of the differentially regulated genes in human $CD4^+$ T cells from three sets of independent studies treated with RAD001, TGB-beta or RAD001 + TGF-beta, of transcriptional changes and translational changes obtained from the ≥ 4 ribosome polysome fraction.

File Name: Supplementary Data 2
Description: Table of ranked quantified changes (increased and decreased) in mRNA expression by TGF-beta treatment, showing transcriptionally altered genes in human $CD4^+$ T cells, providing P-values, log2 ratio changes and major gene functions.

File Name: Supplementary Data 3
Description: Lists of genes corresponding to heatmaps of ranked altered mRNA expression and/or translation represented as log2 fold-changes over untreated controls.

File Name: Supplementary Data 4
Description: Table of ranked quantified transcriptional changes (increased and decreased) in mRNA abundance by RAD001 + TGF-beta treatment, showing transcriptionally altered genes in human $CD4^+$ T cells, providing P-values, log2 ratio changes and major gene functions.

File Name: Supplementary Data 5
Description: Table of ranked quantified changes (increased and decreased) in translatome (mRNA content in ≥4 ribosome polysome fraction) in human $CD4^+$ T cells treated with TGF-beta, showing translationally altered genes, providing P-values, log2 ratio changes and major gene functions.

File Name: Supplementary Data 6
Description: Table of ranked quantified changes (increased and decreased) in translatome (mRNA content in ≥4 ribosome polysome fraction) in human $CD4^+$ T cells treated with RAD001, showing translationally altered genes, providing P-values, log2 ratio changes and major gene functions.

File Name: Supplementary Data 7
Description: Table of ranked quantified changes (increased and decreased) in translatome (mRNA content in ≥4 ribosome polysome fraction) in human $CD4^+$ T cells treated with RAD001 + TGF-beta, showing translationally altered genes, providing P-values, log2 ratio changes and major gene functions.

File Name: Supplementary Data 8
Description: Table of ranked quantified transcriptional changes (increased and decreased) in mRNA abundance by RAD001 treatment, showing transcriptionally altered genes in human $CD4^+$ T cells, providing P-values, log2 ratio changes and major gene functions.
