# Supplementary material for: A DAP5/eIF3d alternate mRNA translation mechanism promotes differentiation and immune suppression by human regulatory T cells
Source: Nat Commun. 2021 Nov 30;12:6979. doi: 10.1038/s41467-021-27087-w (PMC8632918; doi:10.1038/s41467-021-27087-w)
Supplement: Supplementary file 12 — Reporting Summary [file 41467_2021_27087_MOESM12_ESM.pdf]

nature research

Corresponding author(s): Schneider, Robert J.

Last updated by author(s): 10-04-2021

Reporting Summary

Nature Research wishes to improve the reproducibility of the work that we publish. This form provides structure for consistency and transparency in reporting. For further information on Nature Research policies, see our Editorial Policies and the Editorial Policy Checklist.

Statistics

For all statistical analyses, confirm that the following items are present in the figure legend, table legend, main text, or Methods section.

n/a	Confirmed	
☐	☒	The exact sample size (*n*) for each experimental group/condition, given as a discrete number and unit of measurement
☐	☒	A statement on whether measurements were taken from distinct samples or whether the same sample was measured repeatedly
☐	☒	The statistical test(s) used AND whether they are one- or two-sided<br>*Only common tests should be described solely by name; describe more complex techniques in the Methods section.*
☐	☒	A description of all covariates tested
☐	☒	A description of any assumptions or corrections, such as tests of normality and adjustment for multiple comparisons
☐	☒	A full description of the statistical parameters including central tendency (e.g. means) or other basic estimates (e.g. regression coefficient) AND variation (e.g. standard deviation) or associated estimates of uncertainty (e.g. confidence intervals)
☐	☒	For null hypothesis testing, the test statistic (e.g. *F*, *t*, *r*) with confidence intervals, effect sizes, degrees of freedom and *P* value noted<br>*Give P values as exact values whenever suitable.*
☒	☐	For Bayesian analysis, information on the choice of priors and Markov chain Monte Carlo settings
☒	☐	For hierarchical and complex designs, identification of the appropriate level for tests and full reporting of outcomes
☒	☐	Estimates of effect sizes (e.g. Cohen's *d*, Pearson's *r*), indicating how they were calculated

Our web collection on statistics for biologists contains articles on many of the points above.

Software and code

Policy information about availability of computer code

Data collection	UCSC human reference genome: https://genome.ucsc.edu/cgi-bin/hgGateway
Data analysis	MEME data motif analysis suite: https://genome.ucsc.edu/cgi-bin/hgGateway<br>GenomeScope short motif analysis: https://academic.oup.com/bioinformatics/article/33/14/2202/3089939<br>DNAStar SeqBuilder DNA cloning tool: https://www.dnastar.com/software/lasergene/seqbuilder-pro/<br>GraphPad Prism statistical analysis program: https://www.graphpad.com<br>Affymetrix Expression Console software: http://www.affymetrix.com/support/technical/byproduct.affx?product=expressionconsole<br>FlowJo cell FACS and flow cytometry analysis

For manuscripts utilizing custom algorithms or software that are central to the research but not yet described in published literature, software must be made available to editors and reviewers. We strongly encourage code deposition in a community repository (e.g. GitHub). See the Nature Research guidelines for submitting code & software for further information.

Data

Policy information about availability of data

All manuscripts must include a data availability statement. This statement should provide the following information, where applicable:

- Accession codes, unique identifiers, or web links for publicly available datasets
- A list of figures that have associated raw data
- A description of any restrictions on data availability

Data and software availability
Accession numbers for genome-wide data developed in this study have been deposited in GEO under: GSE178634 with hyperlink to the data (https://www.ncbi.nlm.nih.gov/geo/query/acc.cgi?acc=GSE178634). All other data are included in this manuscript s Supplemental data sets 1-3 ; source data for each

display item is provided in the Source Data Excel file Further information and requests for reagents should be directed to and will be fulfilled by the corresponding author, Dr. Robert Schneider (Robert.schneider@nyumc.org). Materials described in this paper are available for distribution under the Uniform Biological Material transfer Agreement, a master agreement that was developed by the NIH to simplify transfers of biological research materials.

Field-specific reporting

Please select the one below that is the best fit for your research. If you are not sure, read the appropriate sections before making your selection.

☒ Life sciences ☐ Behavioural & social sciences ☐ Ecological, evolutionary & environmental sciences

For a reference copy of the document with all sections, see nature.com/documents/nr-reporting-summary-flat.pdf

Life sciences study design

All studies must disclose on these points even when the disclosure is negative.

Sample size	All studies had to produce data that is statistically significant at P<0.05, which determined the number of replicates, with no less than n=3.
Data exclusions	No data was excluded
Replication	Studies were reliably reproduced, tested by experimentally independent replicates. Replicate numbers are described in each figure legend.
Randomization	None of the studies required or involved randomization.
Blinding	There was no need or opportunity for blinded studies in the experiments and analyses that are carried out in this work.

Reporting for specific materials, systems and methods

We require information from authors about some types of materials, experimental systems and methods used in many studies. Here, indicate whether each material, system or method listed is relevant to your study. If you are not sure if a list item applies to your research, read the appropriate section before selecting a response.

Materials & experimental systems

n/a	Involved in the study	
☐	☒	Antibodies
☐	☒	Eukaryotic cell lines
☒	☐	Palaeontology and archaeology
☒	☐	Animals and other organisms
☒	☐	Human research participants
☒	☐	Clinical data
☒	☐	Dual use research of concern

Methods

n/a	Involved in the study	
☒	☐	ChIP-seq
☐	☒	Flow cytometry
☒	☐	MRI-based neuroimaging

Antibodies

Antibodies used	A comprehensive list of antibodies and their sources is provided in the Methods section and as a Supplemental Excel file marked "Antibodies" which describes source, catalogue number and dilutions used.
Validation	Antibodies were all commercial sourced and titrated before use with positive and negative controls.

Eukaryotic cell lines

Policy information about cell lines

Cell line source(s)	ATCC
Authentication	Cell lines were authenticated by STR profiling.
Mycoplasma contamination	All cell lines are tested for mycoplasma on a routine basis, typically every other month
Commonly misidentified lines (See ICLAC register)	N/A

Flow Cytometry

Plots

Confirm that:

☒ The axis labels state the marker and fluorochrome used (e.g. CD4-FITC).

☒ The axis scales are clearly visible. Include numbers along axes only for bottom left plot of group (a 'group' is an analysis of identical markers).

☒ All plots are contour plots with outliers or pseudocolor plots.

☒ A numerical value for number of cells or percentage (with statistics) is provided.

Methodology

Sample preparation	All sample preparation approaches are provided in detail in the Methods section of the manuscript.
Instrument	For flow cytometry this is specified in the Methods section of the manuscript
Software	Several types of software were used for analysis which are specified in the Methods section of the manuscript and above.
Cell population abundance	Where appropriate this is reported int the manuscript
Gating strategy	This is clearly indicated in the manuscript. Gating strategies used, were identified and described in the text and figure legends.

☒ Tick this box to confirm that a figure exemplifying the gating strategy is provided in the Supplementary Information.
