# Supplementary material for: A DAP5/eIF3d alternate mRNA translation mechanism promotes differentiation and immune suppression by human regulatory T cells
Source: Nat Commun. 2021 Nov 30;12:6979. doi: 10.1038/s41467-021-27087-w (PMC8632918; doi:10.1038/s41467-021-27087-w)
Supplement: Supplementary file 13 — Source Data [file 41467_2021_27087_MOESM13_ESM.zip › Source Data/Uncut immunoblots pdf files/Figure 1a immunoblots/030813SSII 001 copy.tif.pdf]

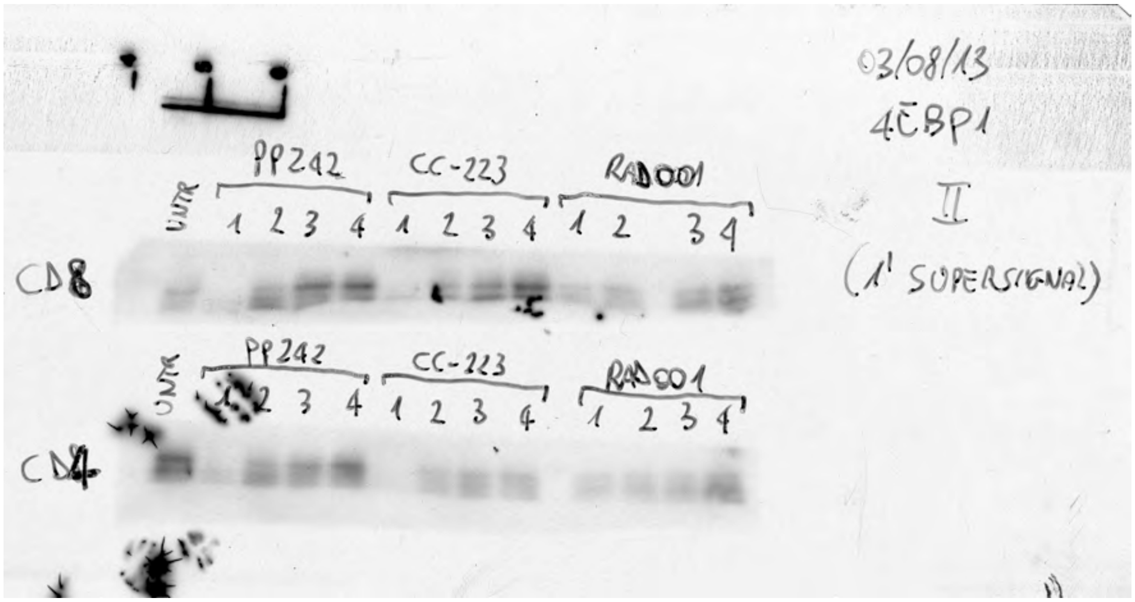
03/08/13
4EBP1
II
(1' SUPERSIGNAL)
CD8
UNTR
PP242
CC-223
RAD001
1 2 3 4
1 2 3 4
1 2 3 4
CD4
UNTR
PP242
CC-223
RAD001
1 2 3 4
1 2 3 4
1 2 3 4
