# Supplementary material for: A DAP5/eIF3d alternate mRNA translation mechanism promotes differentiation and immune suppression by human regulatory T cells
Source: Nat Commun. 2021 Nov 30;12:6979. doi: 10.1038/s41467-021-27087-w (PMC8632918; doi:10.1038/s41467-021-27087-w)
Supplement: Supplementary file 13 — Source Data [file 41467_2021_27087_MOESM13_ESM.zip › Source Data/Uncut immunoblots pdf files/Figure 4e immunoblots/030317_15s copy.tif.pdf]

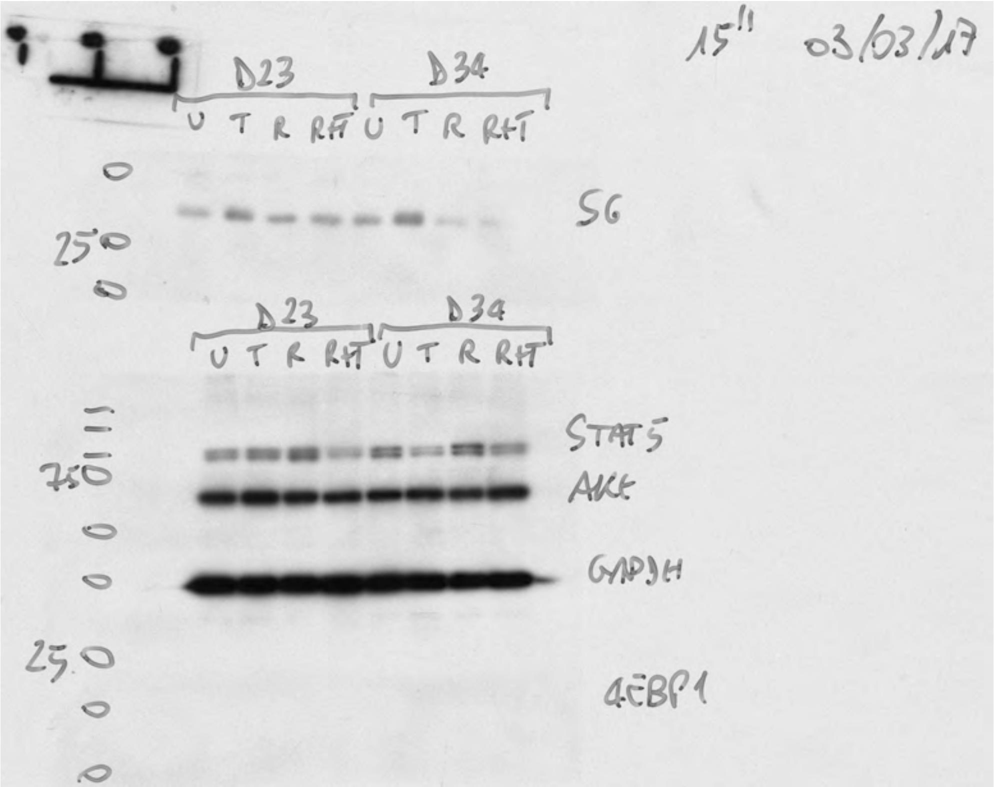
15''
03/03/17
D23
D34
U T R R+T U T R R+T
SG
250
D23
D34
U T R R+T U T R R+T
STAT5
750
AKt
GAPDH
25.0
4EBP1
