# Supplementary figures and images for: A DAP5/eIF3d alternate mRNA translation mechanism promotes differentiation and immune suppression by human regulatory T cells
Source: Nat Commun. 2021 Nov 30;12:6979. doi: 10.1038/s41467-021-27087-w (PMC8632918; doi:10.1038/s41467-021-27087-w)

Uncropped Gels Fig7e

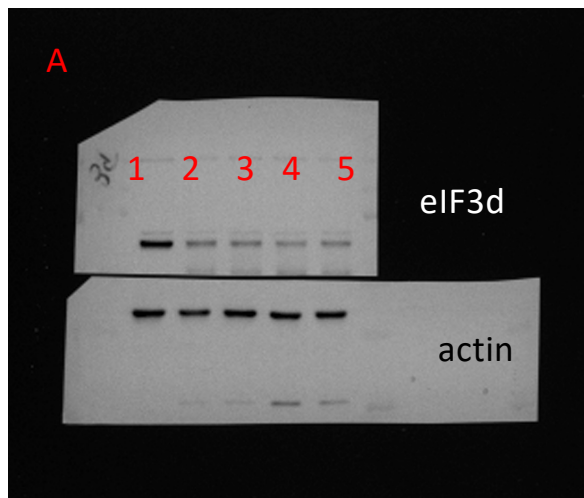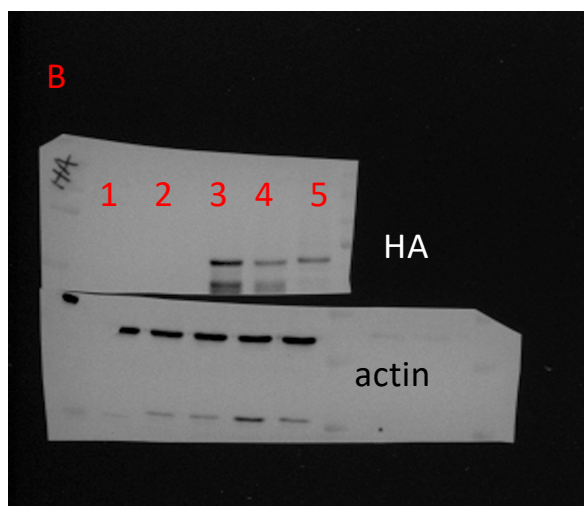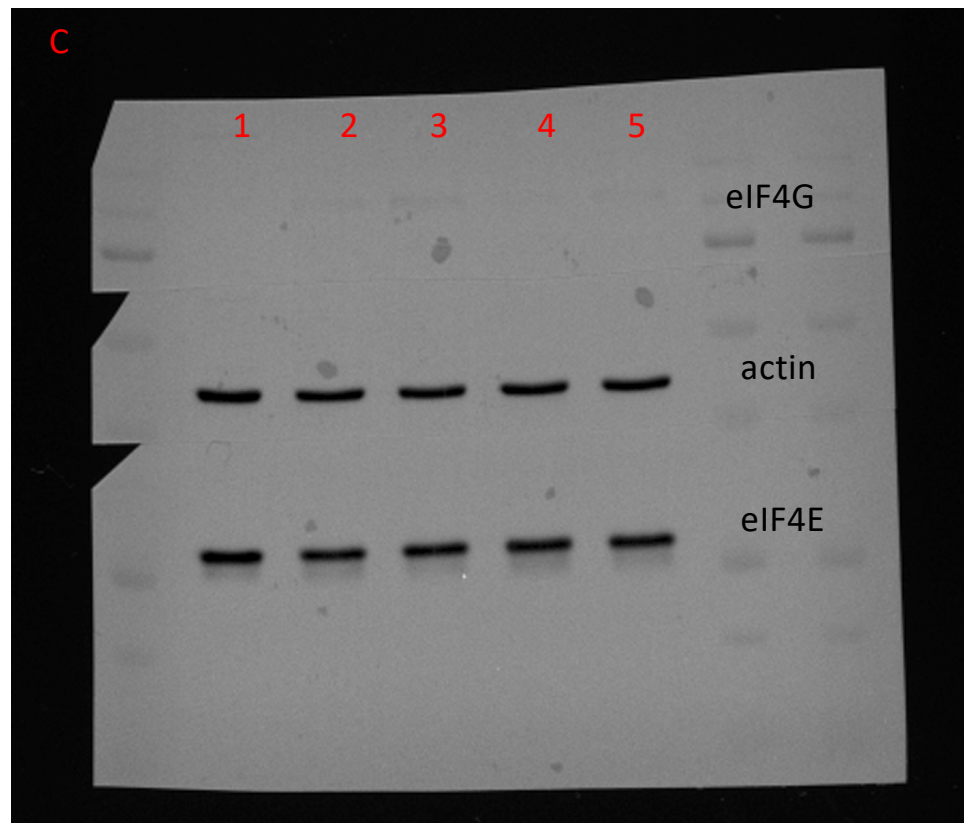

Samples:

1 Nsi

2 bb

3 WT

4 a5

5 a11

Supplement: Supplementary file 13 — Source Data [file 41467_2021_27087_MOESM13_ESM.zip › Source Data/Uncut immunoblots pdf files/Figure 7e immunoblots/Fig7e.pdf]

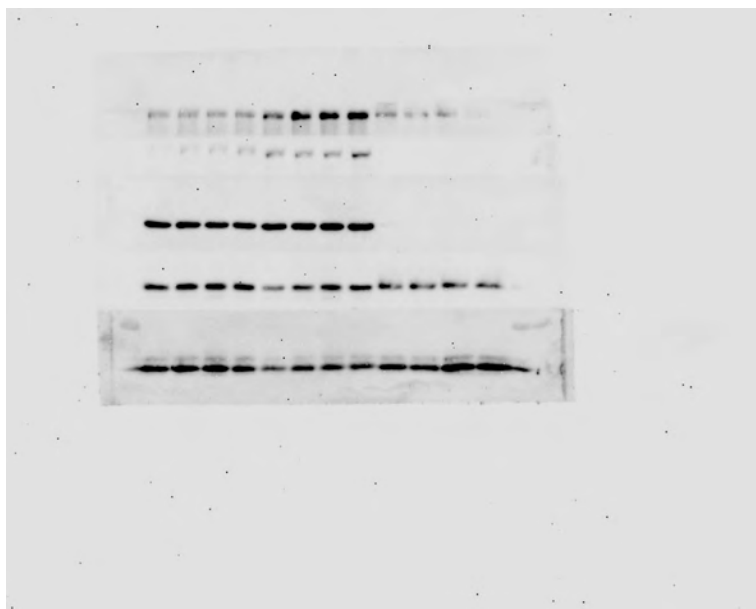

Supplement: Supplementary file 13 — Source Data [file 41467_2021_27087_MOESM13_ESM.zip › Source Data/Uncut immunoblots pdf files/Figure S7a, b immunoblots/2020 02 07 eIF4G, DAP5, b-actin, eIF4E, 4E-BP2.tif.pdf]

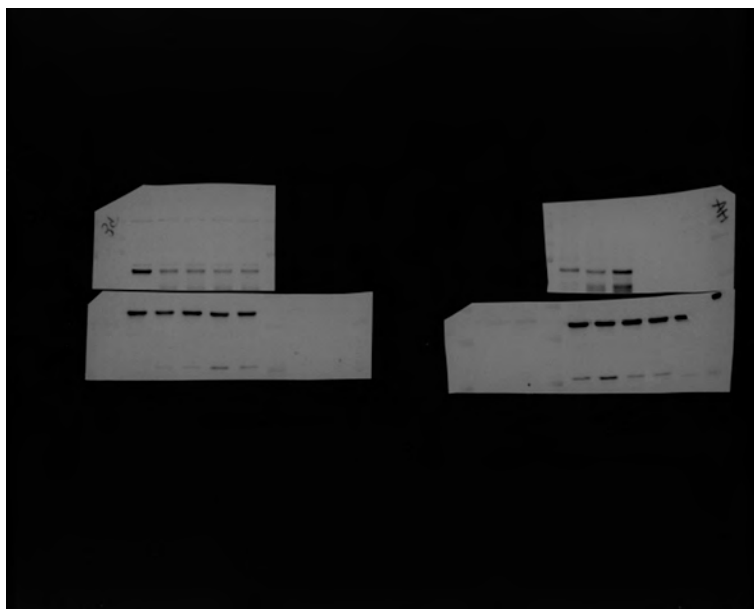

Supplement: Supplementary file 13 — Source Data [file 41467_2021_27087_MOESM13_ESM.zip › Source Data/Uncut immunoblots pdf files/Figure S7a, b immunoblots/2021_03_26_180206.tif.pdf]

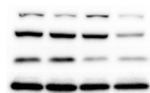

Supplement: Supplementary file 13 — Source Data [file 41467_2021_27087_MOESM13_ESM.zip › Source Data/Uncut immunoblots pdf files/Figure S7a, b immunoblots/2021 01 25 DAP5, eIF3d, eIF4E, GAPDH.tif.pdf]

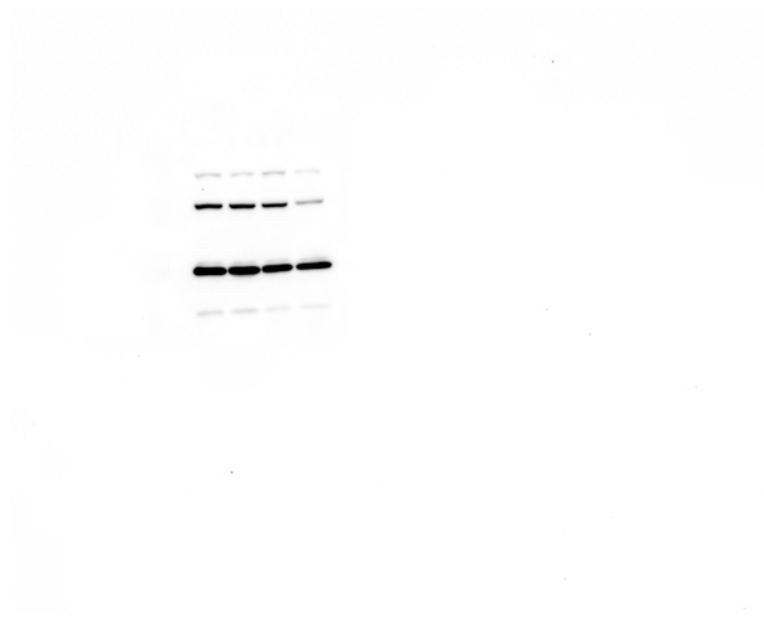

Supplement: Supplementary file 13 — Source Data [file 41467_2021_27087_MOESM13_ESM.zip › Source Data/Uncut immunoblots pdf files/Figure S7a, b immunoblots/2021_01_26_144434.tif.pdf]

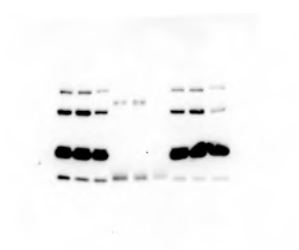

Supplement: Supplementary file 13 — Source Data [file 41467_2021_27087_MOESM13_ESM.zip › Source Data/Uncut immunoblots pdf files/Figure S7a, b immunoblots/2021_02_09_114852.tif.pdf]

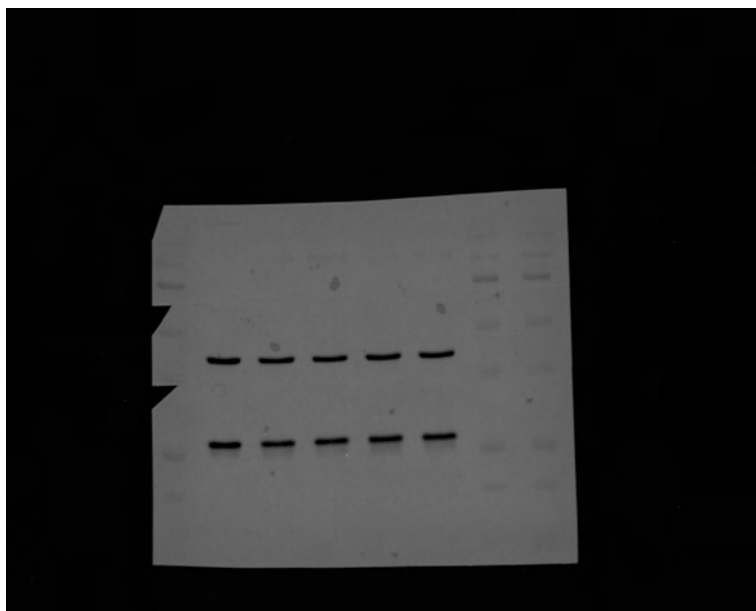

Supplement: Supplementary file 13 — Source Data [file 41467_2021_27087_MOESM13_ESM.zip › Source Data/Uncut immunoblots pdf files/Figure S7a, b immunoblots/4eactinvv_olay.tif.pdf]

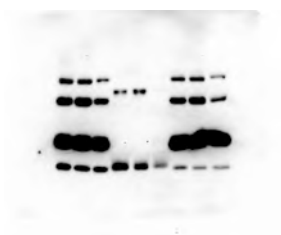

Supplement: Supplementary file 13 — Source Data [file 41467_2021_27087_MOESM13_ESM.zip › Source Data/Uncut immunoblots pdf files/Figure S7a, b immunoblots/2021_02_09_114938.tif.pdf]

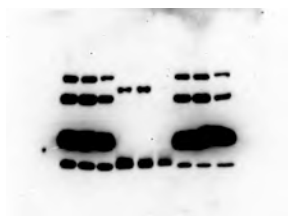

Supplement: Supplementary file 13 — Source Data [file 41467_2021_27087_MOESM13_ESM.zip › Source Data/Uncut immunoblots pdf files/Figure S7a, b immunoblots/2021_02_09_114044.tif.pdf]

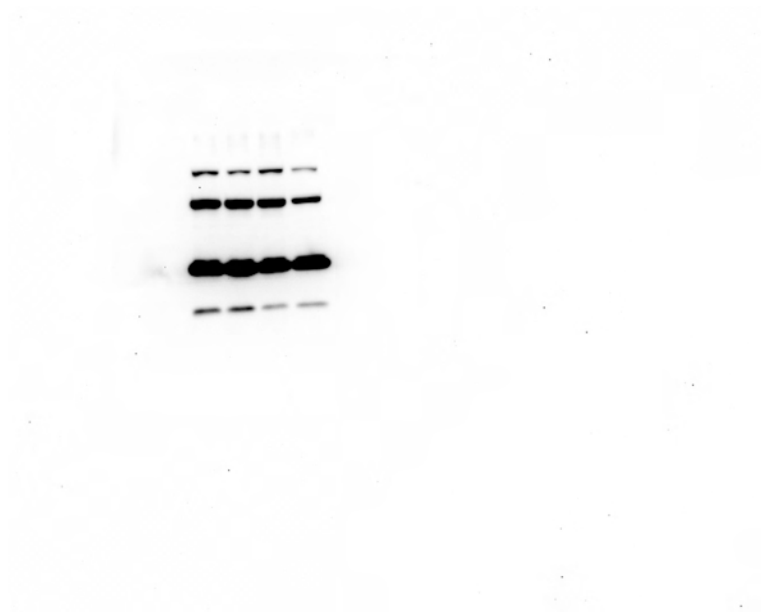

Supplement: Supplementary file 13 — Source Data [file 41467_2021_27087_MOESM13_ESM.zip › Source Data/Uncut immunoblots pdf files/Figure S7a, b immunoblots/2021_01_26_144616.tif.pdf]

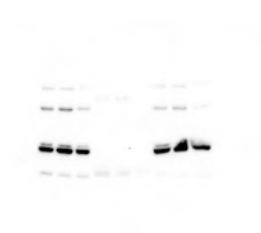

Supplement: Supplementary file 13 — Source Data [file 41467_2021_27087_MOESM13_ESM.zip › Source Data/Uncut immunoblots pdf files/Figure S7a, b immunoblots/2021_02_09_114805.tif.pdf]

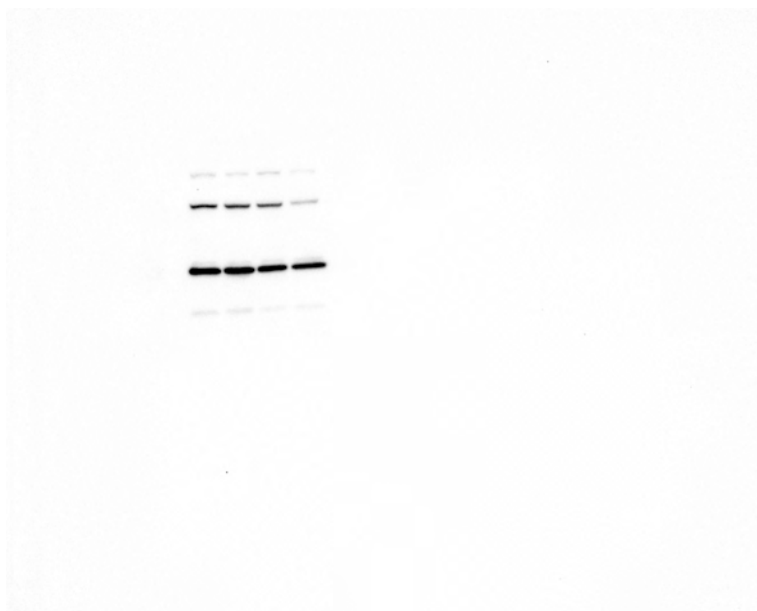

Supplement: Supplementary file 13 — Source Data [file 41467_2021_27087_MOESM13_ESM.zip › Source Data/Uncut immunoblots pdf files/Figure S7a, b immunoblots/2021_01_26_144538.tif.pdf]

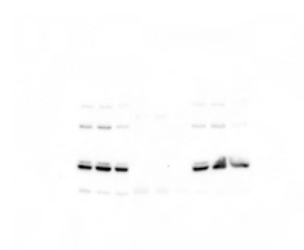

Supplement: Supplementary file 13 — Source Data [file 41467_2021_27087_MOESM13_ESM.zip › Source Data/Uncut immunoblots pdf files/Figure S7a, b immunoblots/2021_02_09_114728.tif.pdf]

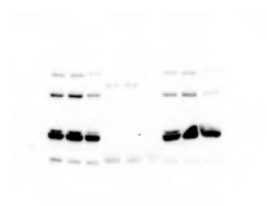

Supplement: Supplementary file 13 — Source Data [file 41467_2021_27087_MOESM13_ESM.zip › Source Data/Uncut immunoblots pdf files/Figure S7a, b immunoblots/2021_02_09_114826.tif.pdf]

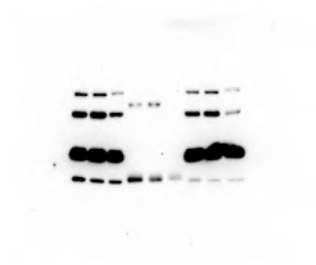

Supplement: Supplementary file 13 — Source Data [file 41467_2021_27087_MOESM13_ESM.zip › Source Data/Uncut immunoblots pdf files/Figure S7a, b immunoblots/2021_02_09_113909.tif.pdf]

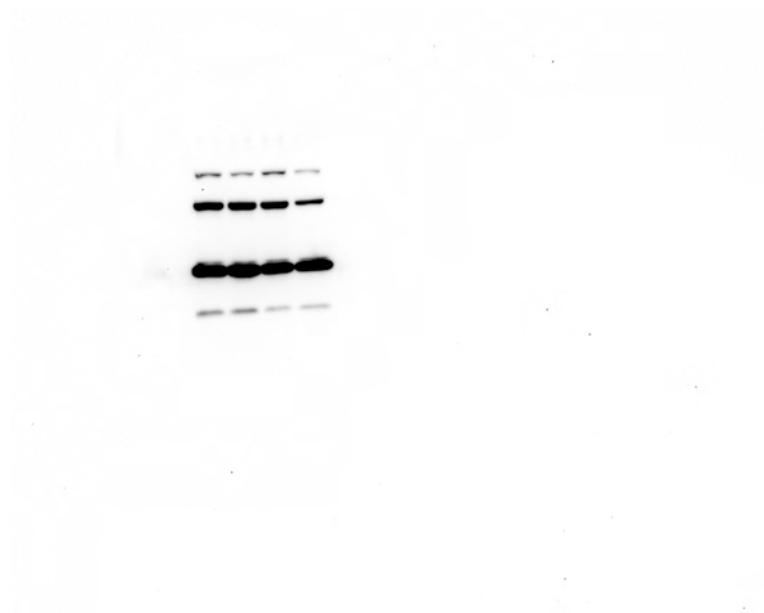

Supplement: Supplementary file 13 — Source Data [file 41467_2021_27087_MOESM13_ESM.zip › Source Data/Uncut immunoblots pdf files/Figure S7a, b immunoblots/2021_01_26_144504.tif.pdf]

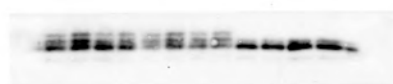

Supplement: Supplementary file 13 — Source Data [file 41467_2021_27087_MOESM13_ESM.zip › Source Data/Uncut immunoblots pdf files/Figure S7a, b immunoblots/2020 02 12 4E-BP1.tif.pdf]

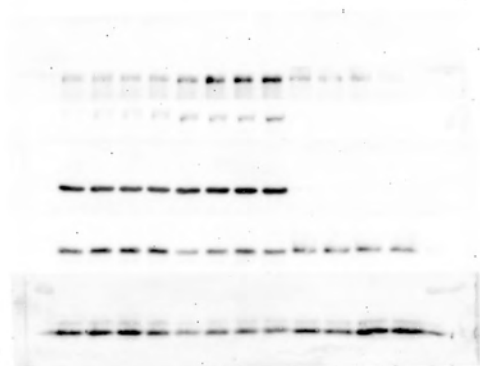

Supplement: Supplementary file 13 — Source Data [file 41467_2021_27087_MOESM13_ESM.zip › Source Data/Uncut immunoblots pdf files/Figure 7a immunoblots/2020 02 07 eIF4G, DAP5, b-actin, eIF4E, 4E-BP2.tif.pdf]

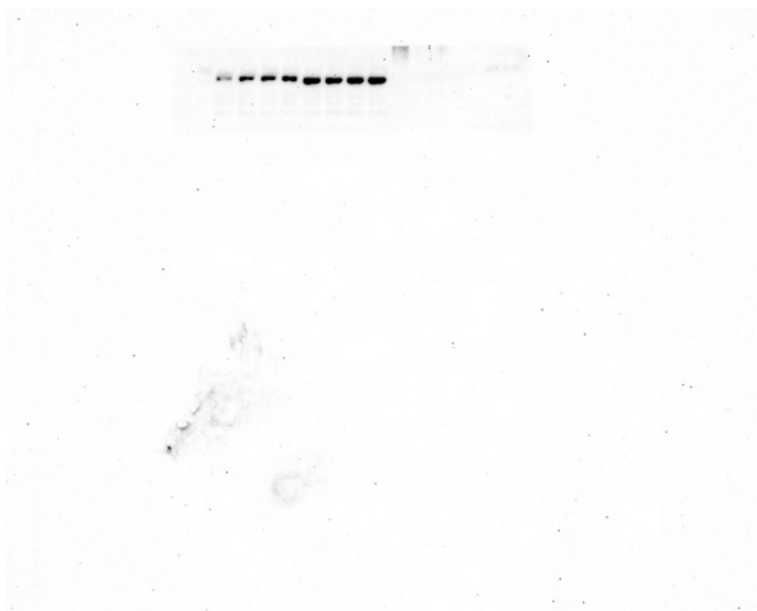

Supplement: Supplementary file 13 — Source Data [file 41467_2021_27087_MOESM13_ESM.zip › Source Data/Uncut immunoblots pdf files/Figure 7a immunoblots/2020 09 30 eIF3d.tif.pdf]

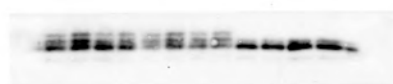

Supplement: Supplementary file 13 — Source Data [file 41467_2021_27087_MOESM13_ESM.zip › Source Data/Uncut immunoblots pdf files/Figure 7a immunoblots/2020 02 12 4E-BP1.tif.pdf]

03/08/13

BRSA

30''

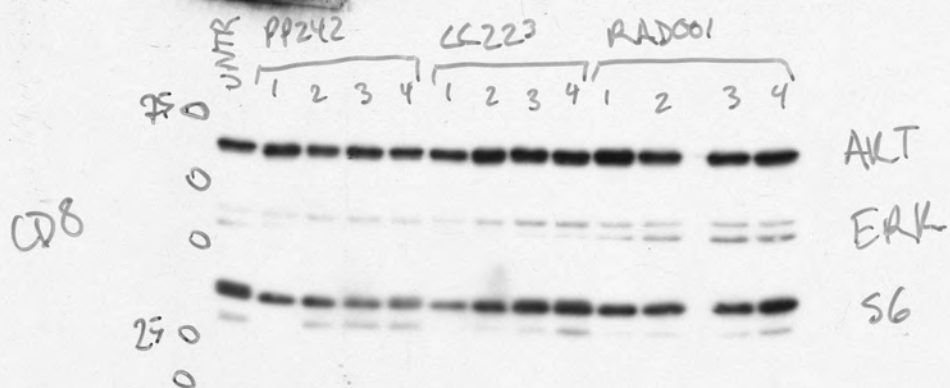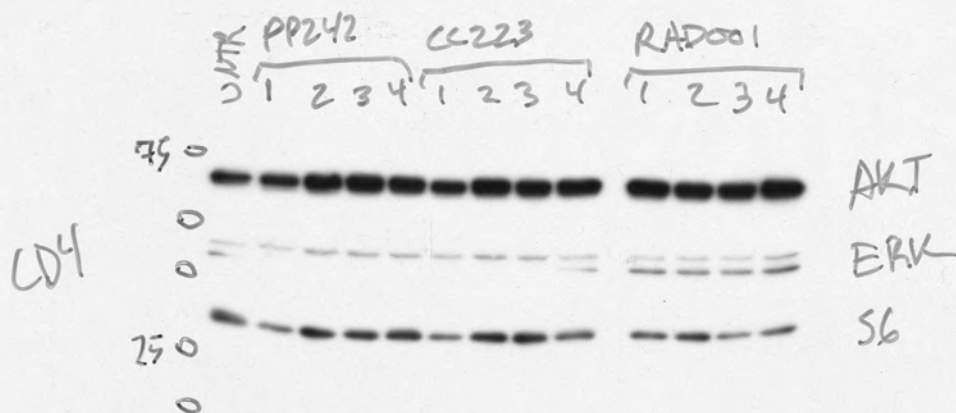

Supplement: Supplementary file 13 — Source Data [file 41467_2021_27087_MOESM13_ESM.zip › Source Data/Uncut immunoblots pdf files/Figure 1a immunoblots/030813 30s copy.tif.pdf]

03/08/13

~~11~~  
11

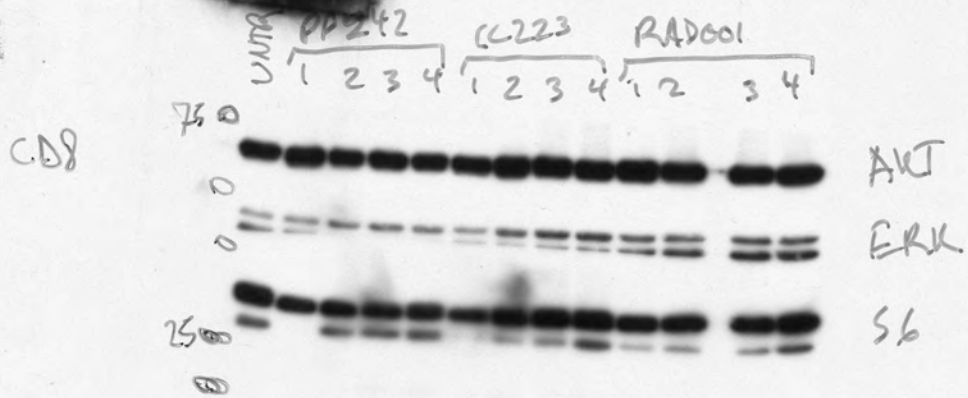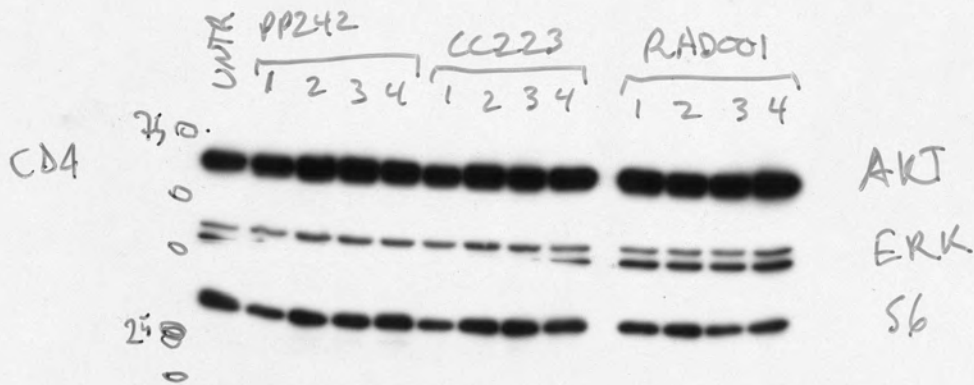

Supplement: Supplementary file 13 — Source Data [file 41467_2021_27087_MOESM13_ESM.zip › Source Data/Uncut immunoblots pdf files/Figure 1a immunoblots/030813 1m copy.tif.pdf]

03/08/13

11

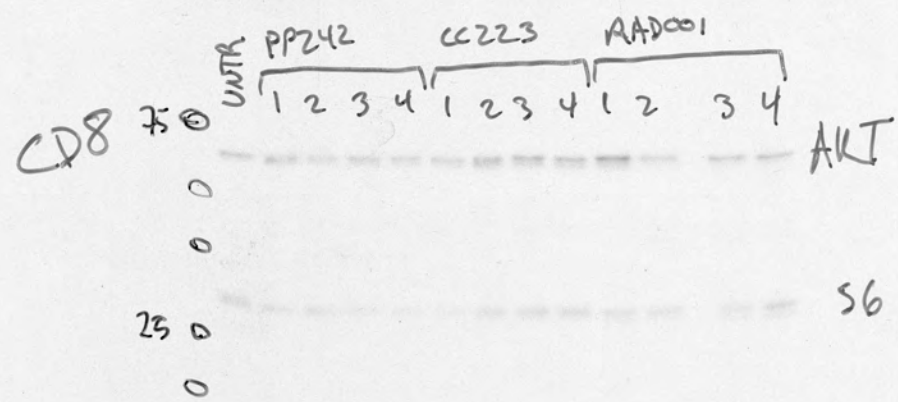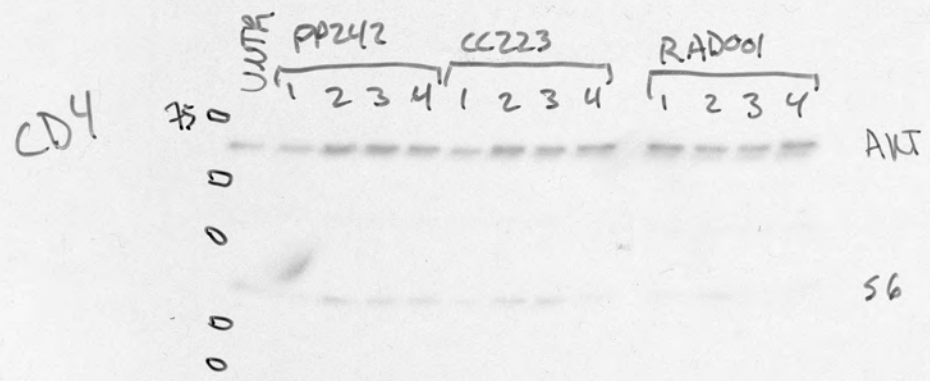

Supplement: Supplementary file 13 — Source Data [file 41467_2021_27087_MOESM13_ESM.zip › Source Data/Uncut immunoblots pdf files/Figure 1a immunoblots/030813 1s copy.tif.pdf]

03/08/13  
15"

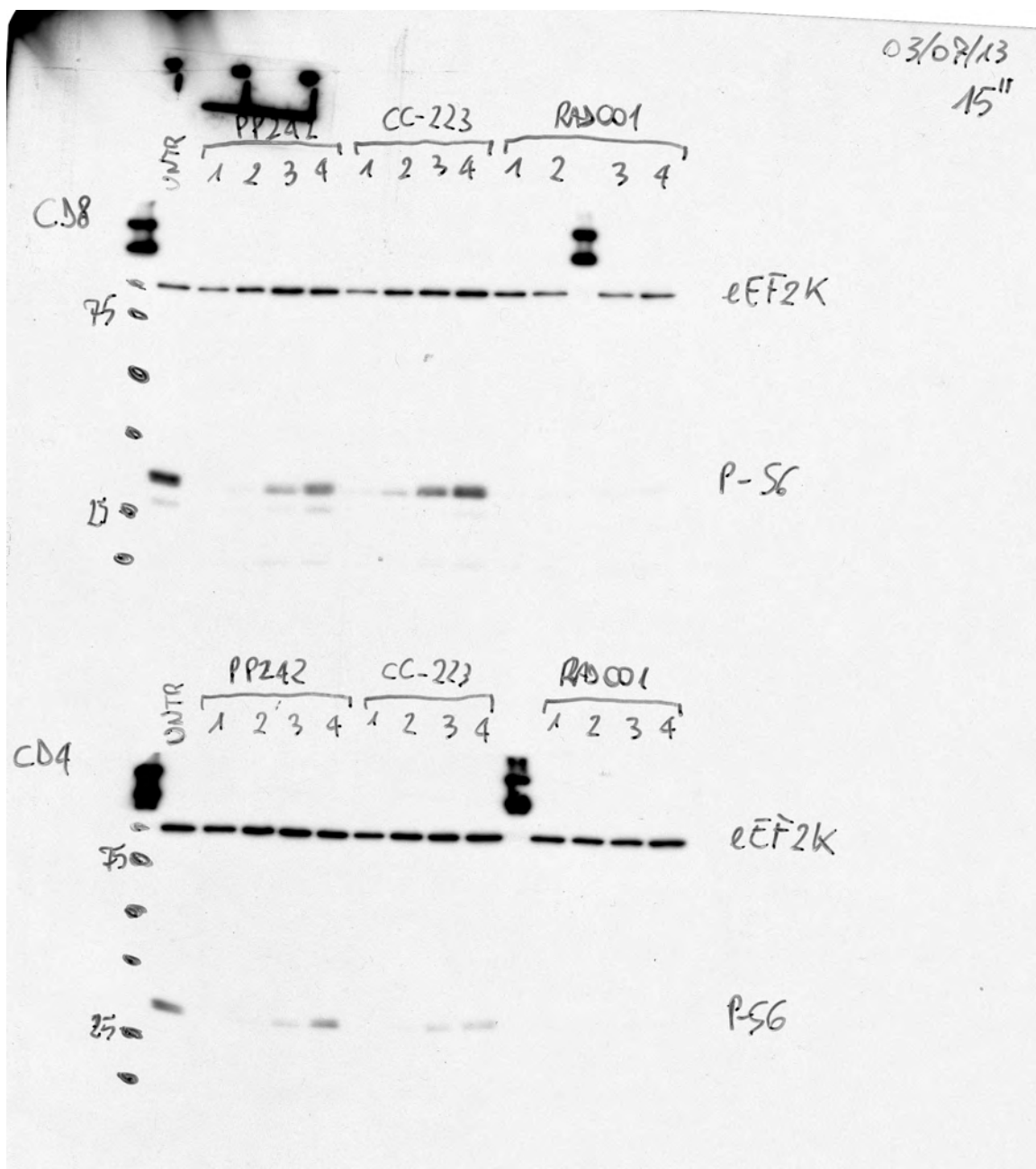

Supplement: Supplementary file 13 — Source Data [file 41467_2021_27087_MOESM13_ESM.zip › Source Data/Uncut immunoblots pdf files/Figure 1a immunoblots/030713 15s copy.tif.pdf]

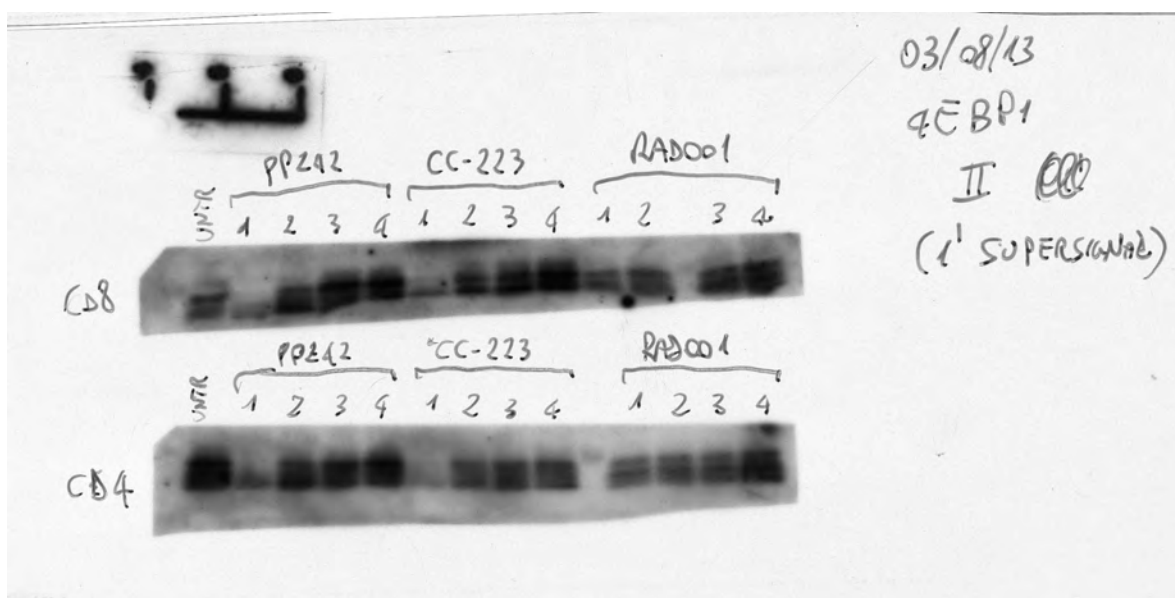

Supplement: Supplementary file 13 — Source Data [file 41467_2021_27087_MOESM13_ESM.zip › Source Data/Uncut immunoblots pdf files/Figure 1a immunoblots/030813SSII 003 copy.tif.pdf]

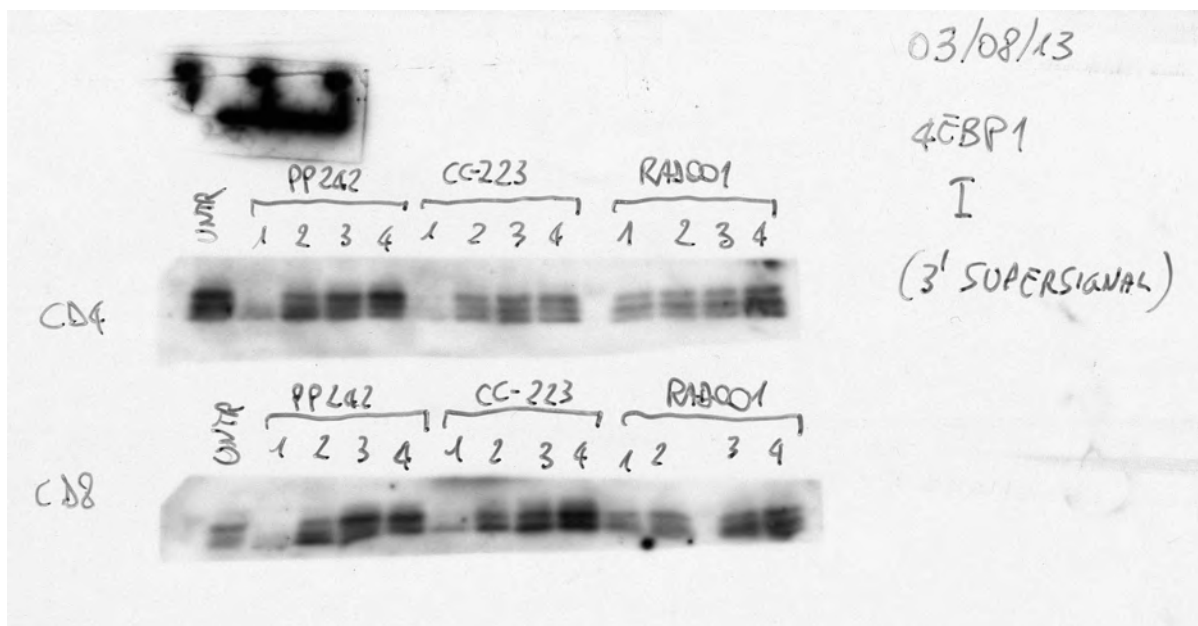

Supplement: Supplementary file 13 — Source Data [file 41467_2021_27087_MOESM13_ESM.zip › Source Data/Uncut immunoblots pdf files/Figure 1a immunoblots/030813SSI 003 copy.tif.pdf]

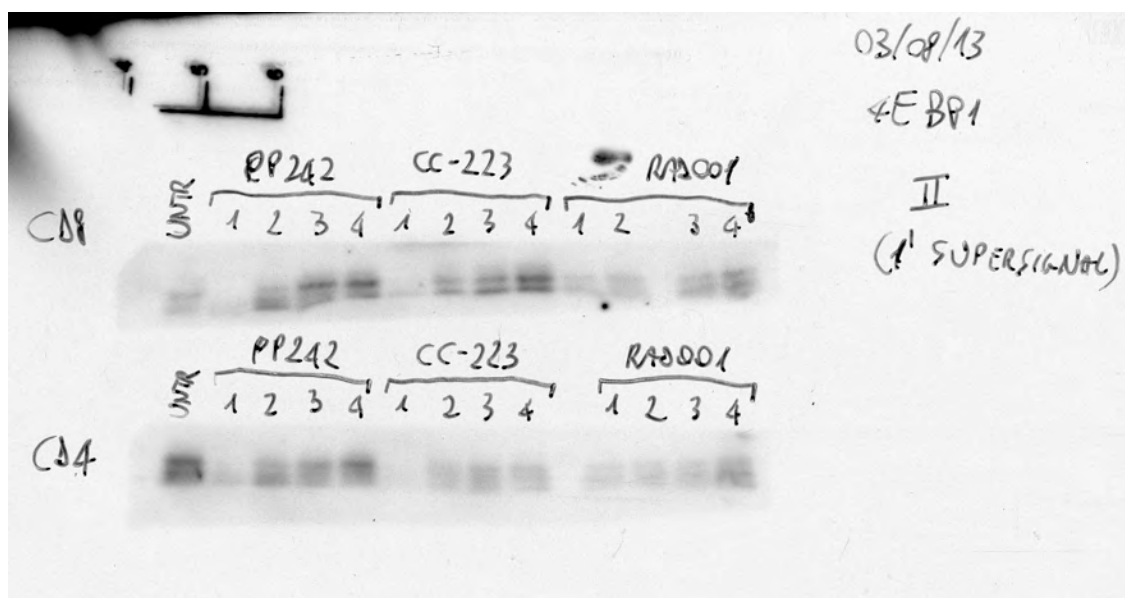

Supplement: Supplementary file 13 — Source Data [file 41467_2021_27087_MOESM13_ESM.zip › Source Data/Uncut immunoblots pdf files/Figure 1a immunoblots/030813SSII 002 copy.tif.pdf]

03/08/13

10"

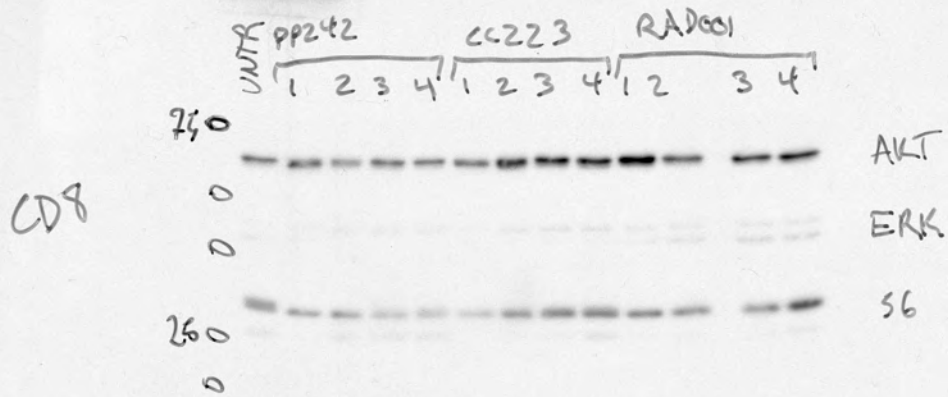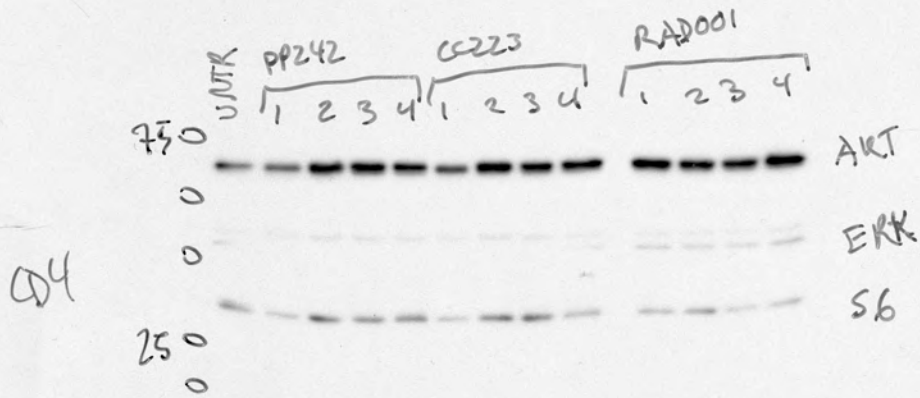

Supplement: Supplementary file 13 — Source Data [file 41467_2021_27087_MOESM13_ESM.zip › Source Data/Uncut immunoblots pdf files/Figure 1a immunoblots/030813 10s copy.tif.pdf]

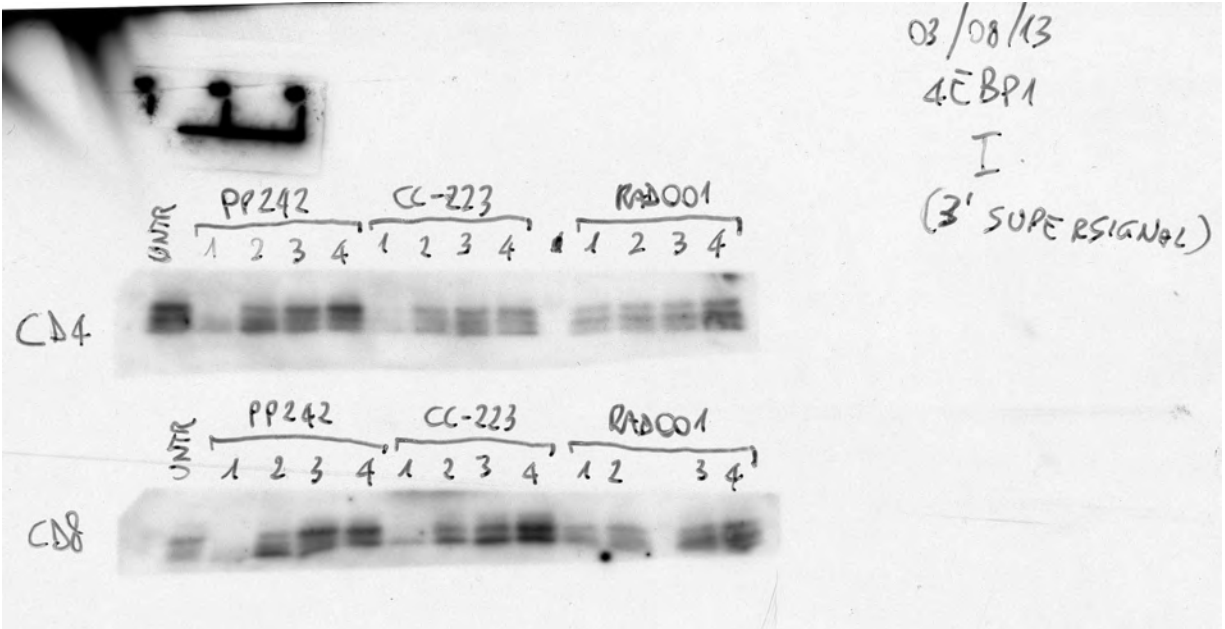

Supplement: Supplementary file 13 — Source Data [file 41467_2021_27087_MOESM13_ESM.zip › Source Data/Uncut immunoblots pdf files/Figure 1a immunoblots/030813SSI 002 copy.tif.pdf]

03/08/13

BBN 11  
15

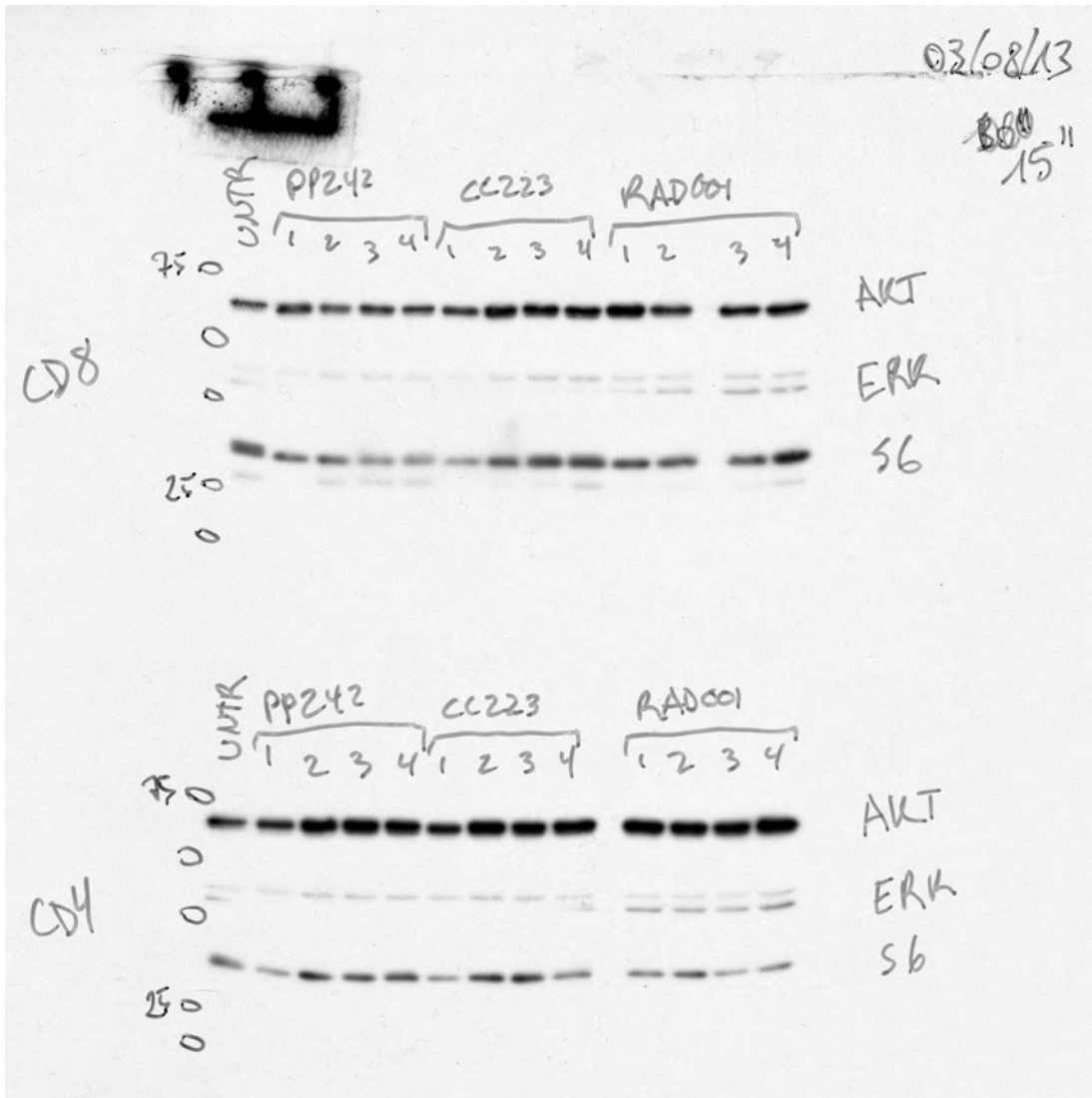

Supplement: Supplementary file 13 — Source Data [file 41467_2021_27087_MOESM13_ESM.zip › Source Data/Uncut immunoblots pdf files/Figure 1a immunoblots/030813 15s copy.tif.pdf]

03/07/13  
5'

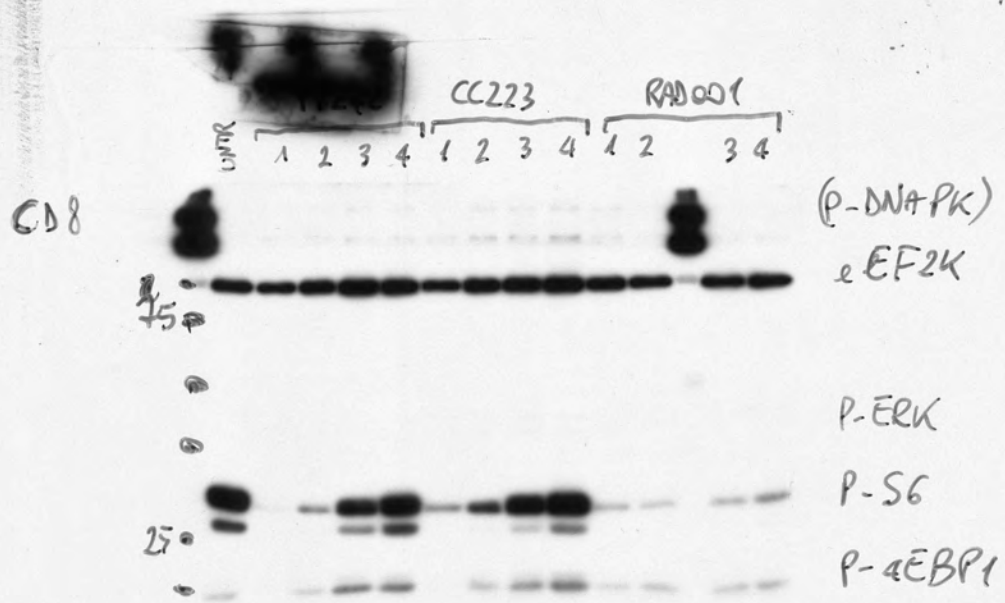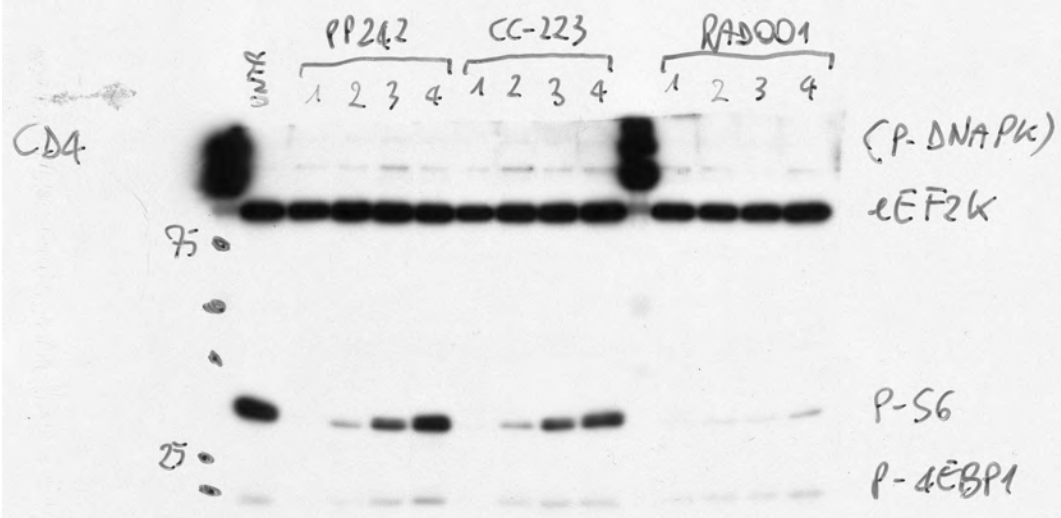

Supplement: Supplementary file 13 — Source Data [file 41467_2021_27087_MOESM13_ESM.zip › Source Data/Uncut immunoblots pdf files/Figure 1a immunoblots/030713 5m copy.tif.pdf]

03/07/13

10'

CD8

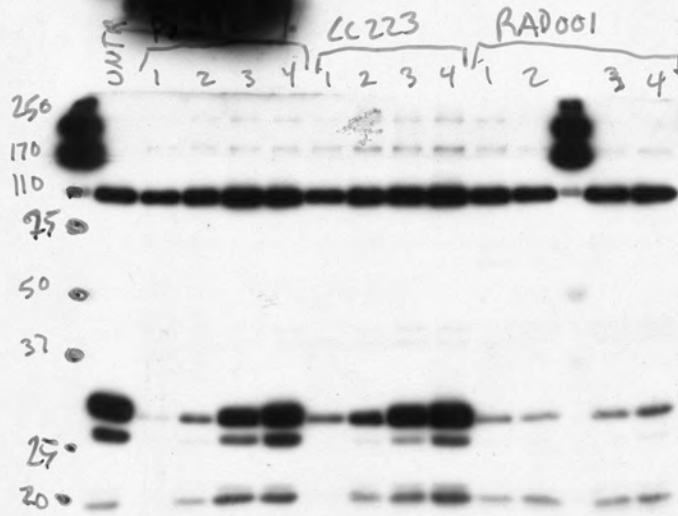

(P-DNA-PK)  
eEF2K  
P-AKT S473  
P-ERK  
P-S6  
P-4EBP

CD4

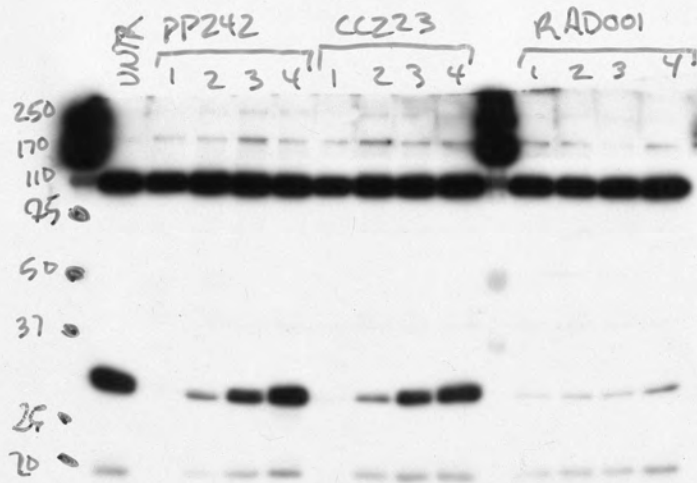

(P-DNA-PK)  
eEF2K  
P-AKT S473  
P-ERK  
P-S6  
P-4EBP

Supplement: Supplementary file 13 — Source Data [file 41467_2021_27087_MOESM13_ESM.zip › Source Data/Uncut immunoblots pdf files/Figure 1a immunoblots/030713 10m copy.tif.pdf]

03/07/13

1'

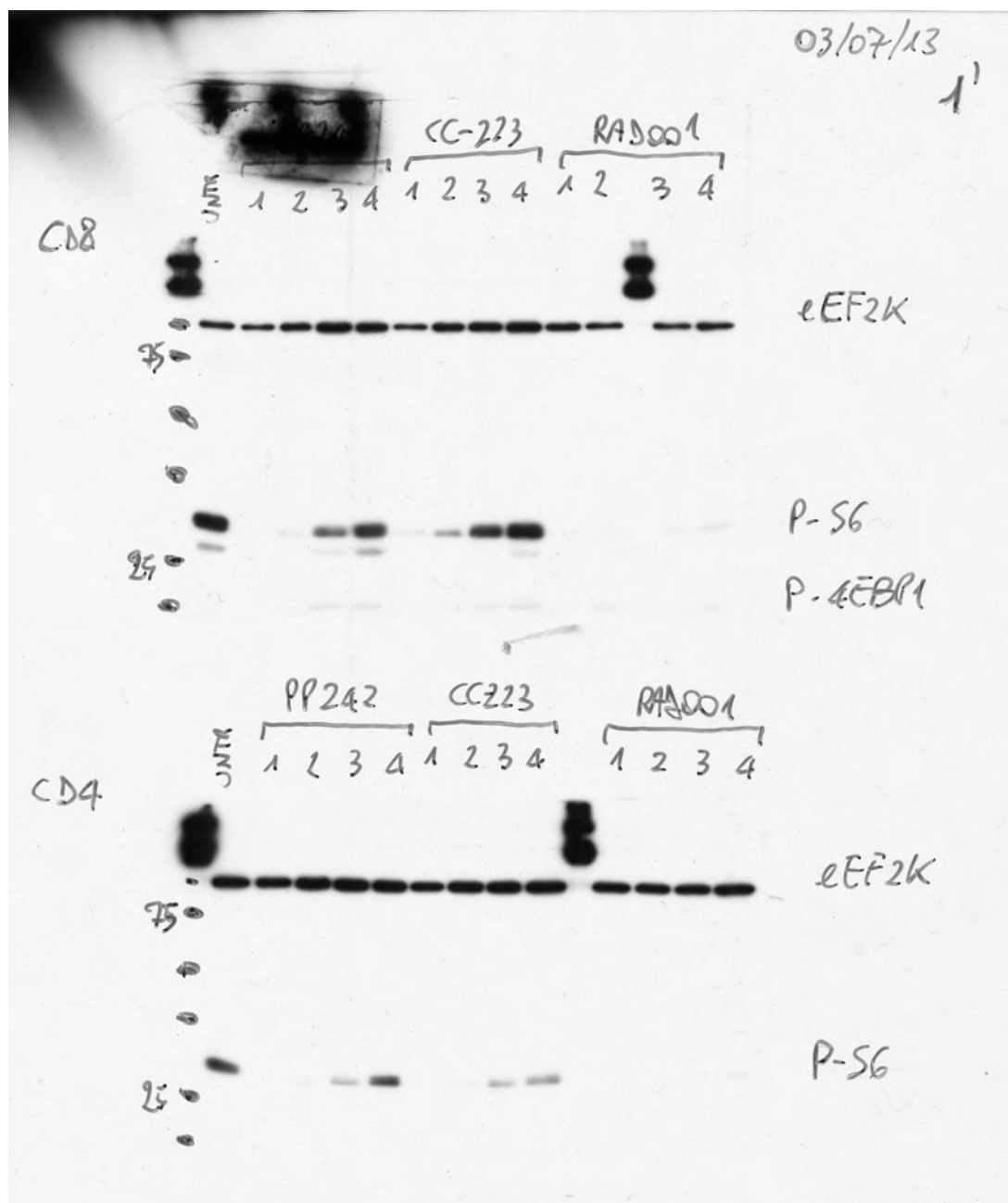

Supplement: Supplementary file 13 — Source Data [file 41467_2021_27087_MOESM13_ESM.zip › Source Data/Uncut immunoblots pdf files/Figure 1a immunoblots/030713 1m copy.tif.pdf]

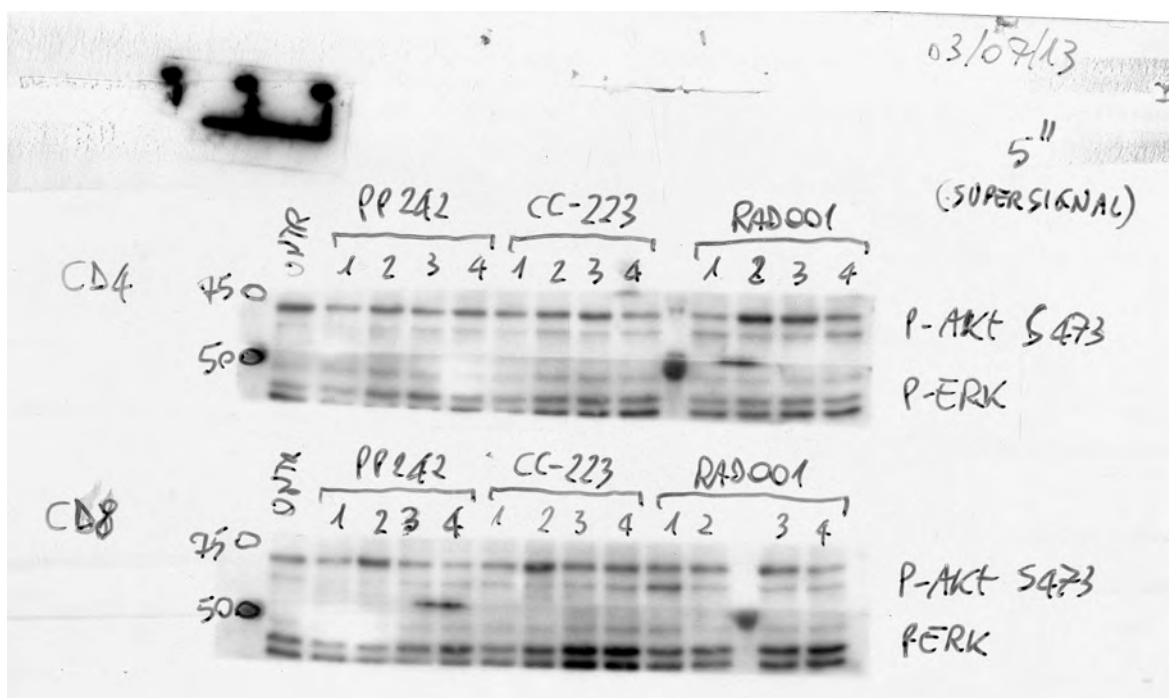

Supplement: Supplementary file 13 — Source Data [file 41467_2021_27087_MOESM13_ESM.zip › Source Data/Uncut immunoblots pdf files/Figure 1a immunoblots/030713SS 5s copy.tif.pdf]

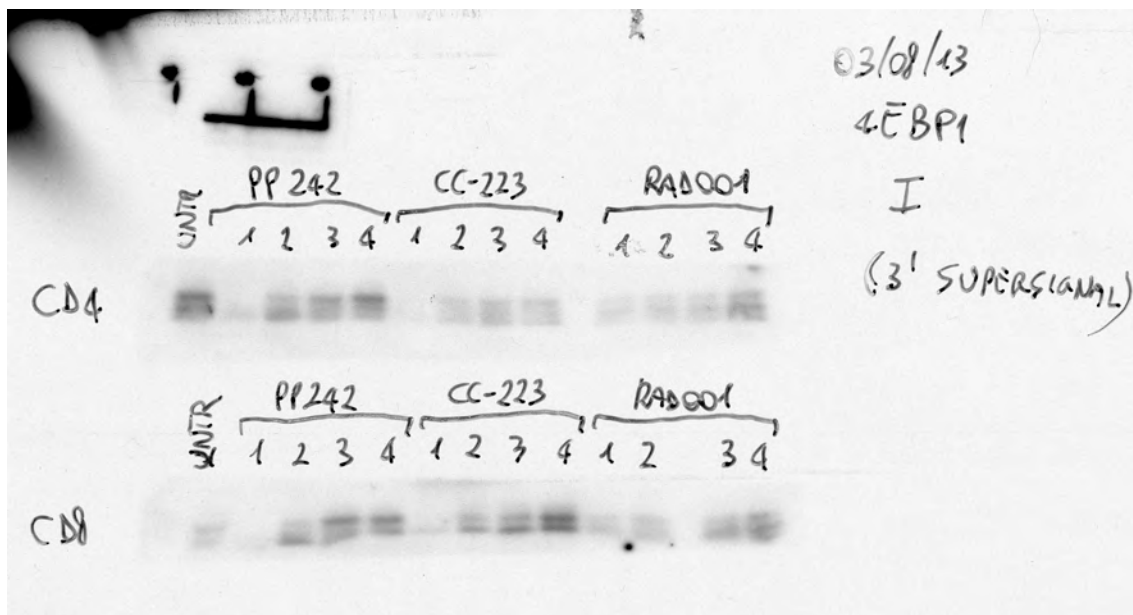

Supplement: Supplementary file 13 — Source Data [file 41467_2021_27087_MOESM13_ESM.zip › Source Data/Uncut immunoblots pdf files/Figure 1a immunoblots/030813SSI 001 copy.tif.pdf]

03/07/13

10<sup>11</sup>  
(SUPER SIGNAL)

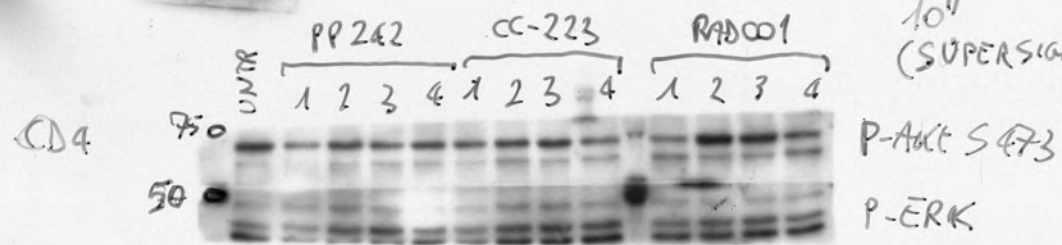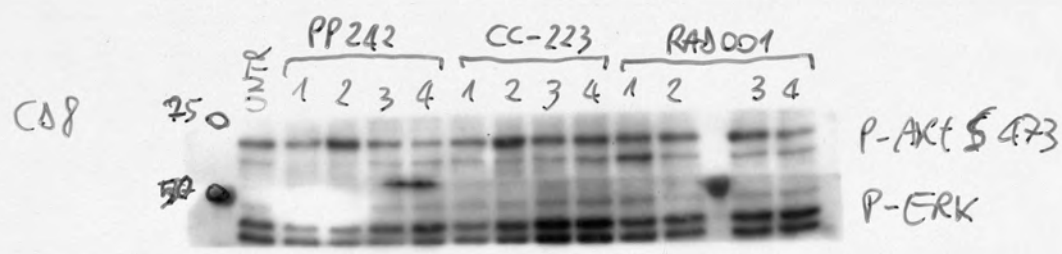

Supplement: Supplementary file 13 — Source Data [file 41467_2021_27087_MOESM13_ESM.zip › Source Data/Uncut immunoblots pdf files/Figure 1a immunoblots/030713SS 10s copy.tif.pdf]

03/07/13

1''

(SUPER SENSITIVE)

CD4

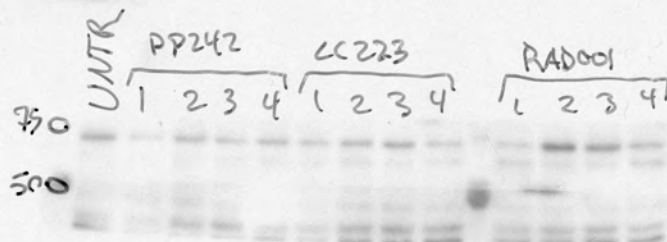

P-AKT S473

P-ERK

CD8

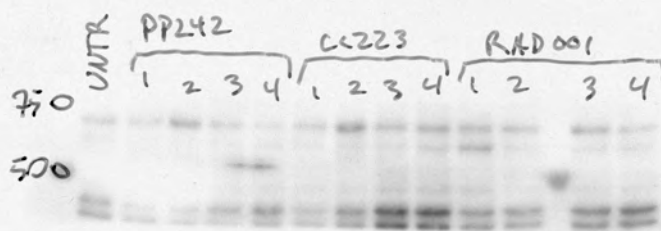

P-AKT S473

P-ERK

Supplement: Supplementary file 13 — Source Data [file 41467_2021_27087_MOESM13_ESM.zip › Source Data/Uncut immunoblots pdf files/Figure 1a immunoblots/030713SS 1s copy.tif.pdf]

3011 03/07/13

(SUPERSONAL)

CD4

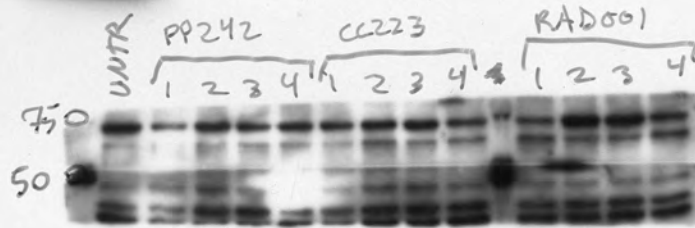

P-AKT S473

P-ERK

CD8

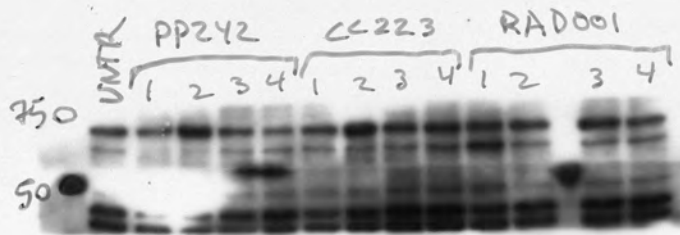

P-AKT S473

P-ERK

Supplement: Supplementary file 13 — Source Data [file 41467_2021_27087_MOESM13_ESM.zip › Source Data/Uncut immunoblots pdf files/Figure 1a immunoblots/030713SS 30s copy.tif.pdf]

03/07/13

30''

CD8

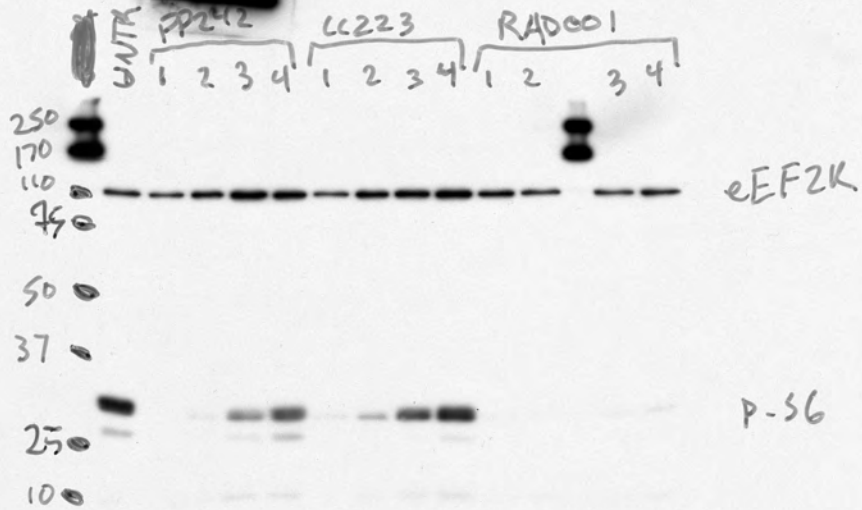

CD4

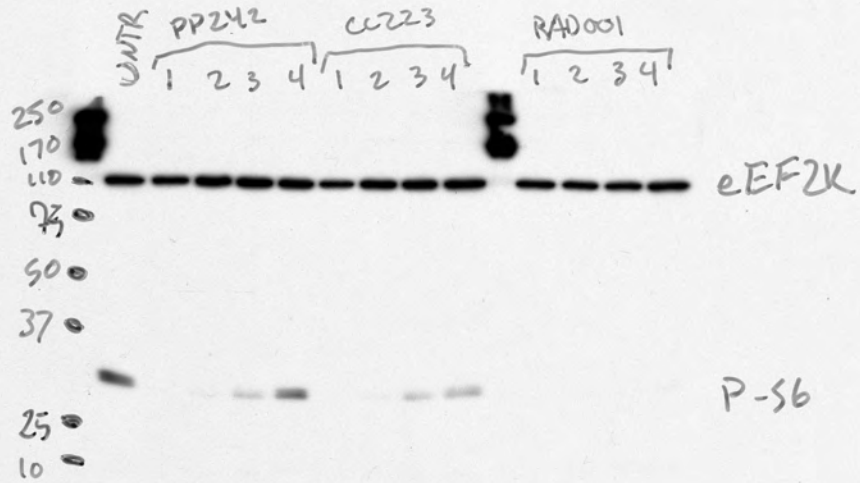

Supplement: Supplementary file 13 — Source Data [file 41467_2021_27087_MOESM13_ESM.zip › Source Data/Uncut immunoblots pdf files/Figure 1a immunoblots/030713 30s copy.tif.pdf]

08/07/13

30'

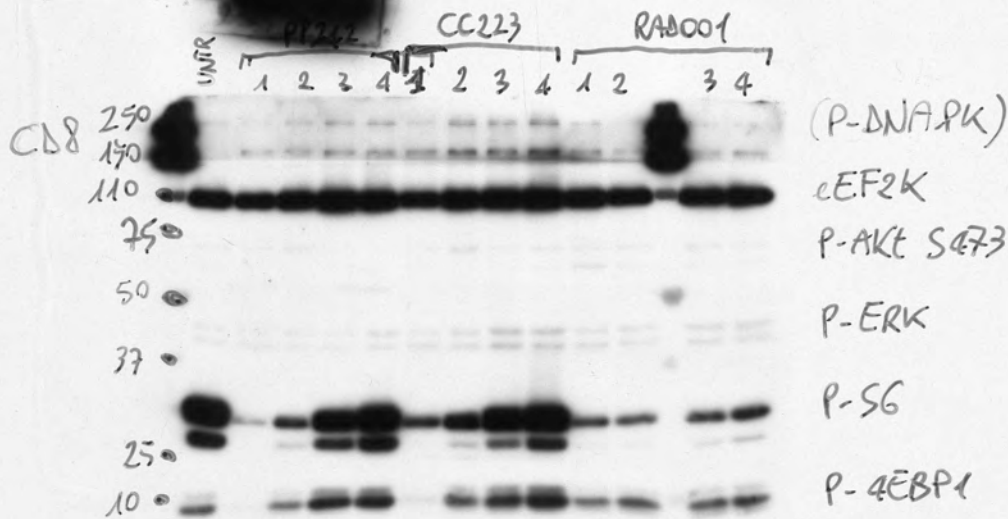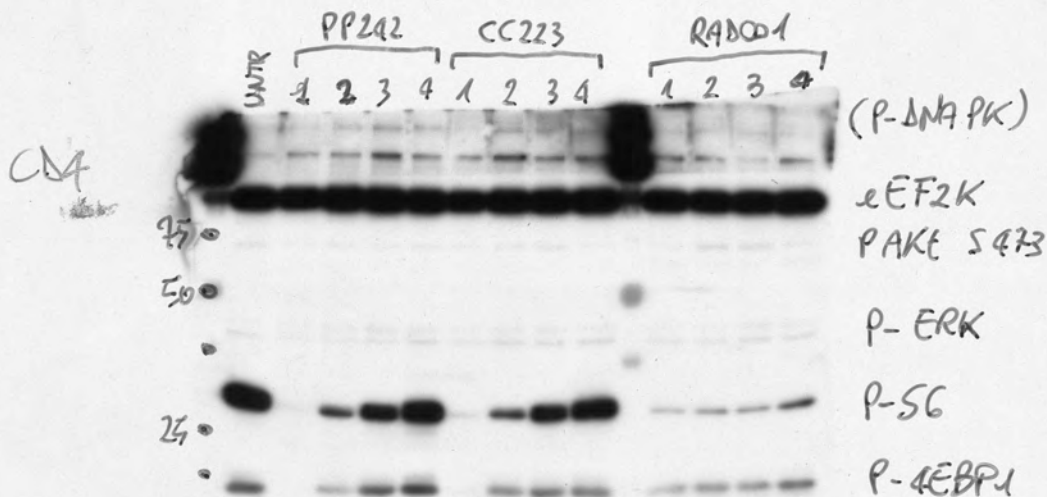

Supplement: Supplementary file 13 — Source Data [file 41467_2021_27087_MOESM13_ESM.zip › Source Data/Uncut immunoblots pdf files/Figure 1a immunoblots/030713 30m copy.tif.pdf]

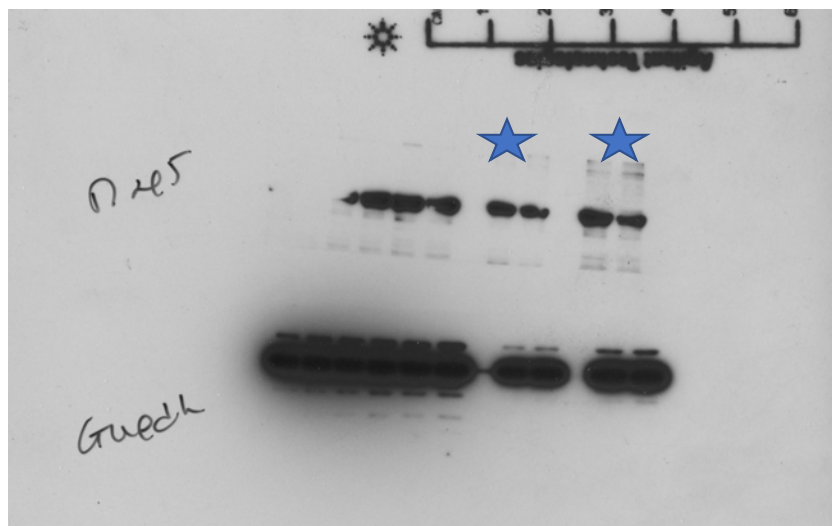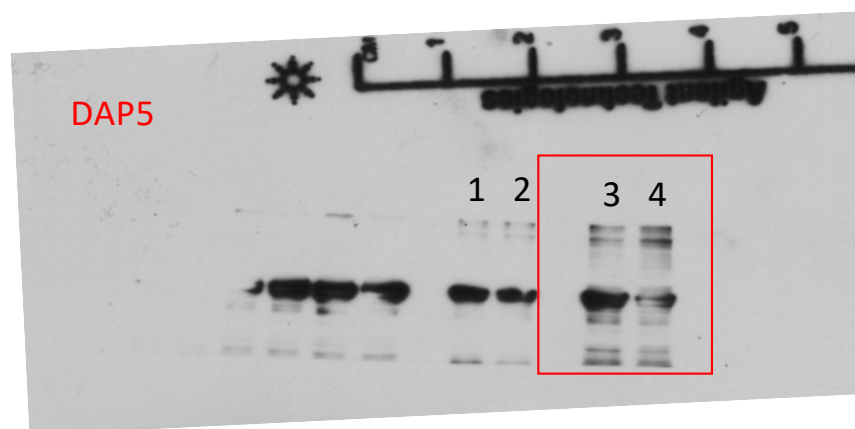

Samples:  
1 Nsi 7ug  
2 shDAP5 7ug  
3 Nsi 15ug  
4 shDAP5 15ug

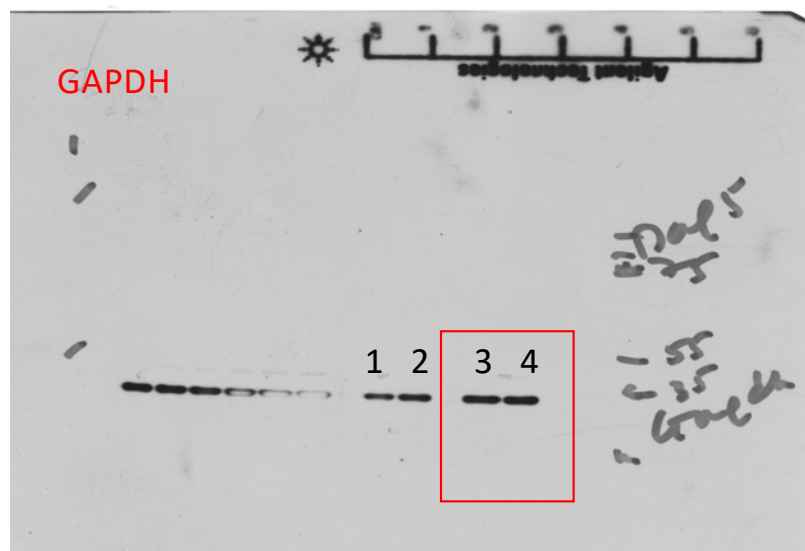

Supplement: Supplementary file 13 — Source Data [file 41467_2021_27087_MOESM13_ESM.zip › Source Data/Uncut immunoblots pdf files/Figure 7d immunoblots/original WB of IVT_shDAP5.pdf]

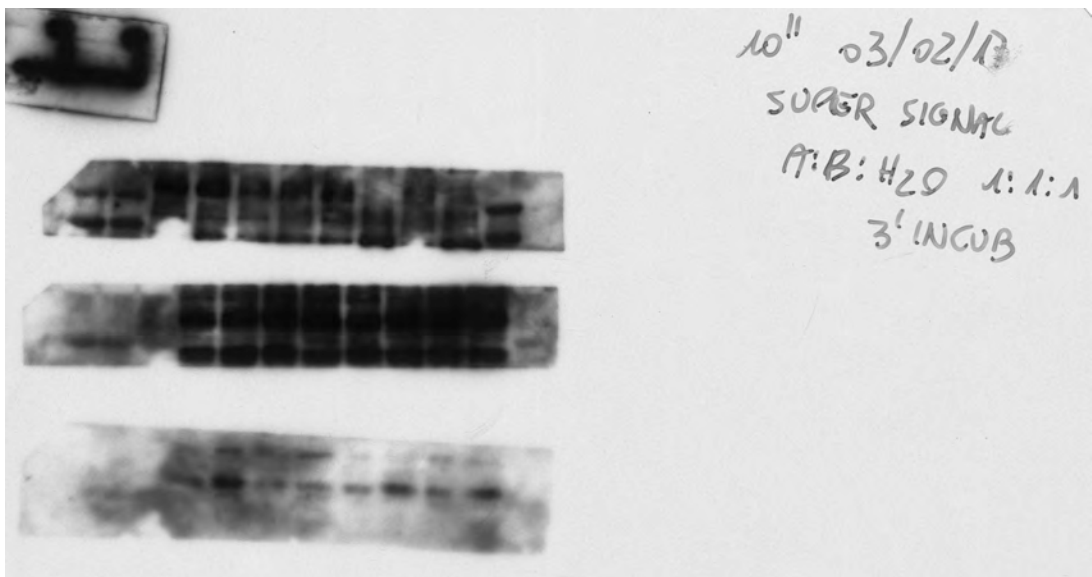

Supplement: Supplementary file 13 — Source Data [file 41467_2021_27087_MOESM13_ESM.zip › Source Data/Uncut immunoblots pdf files/Figure 4e immunoblots/030217_SS_10s copy.tif.pdf]

30" 03/2/17

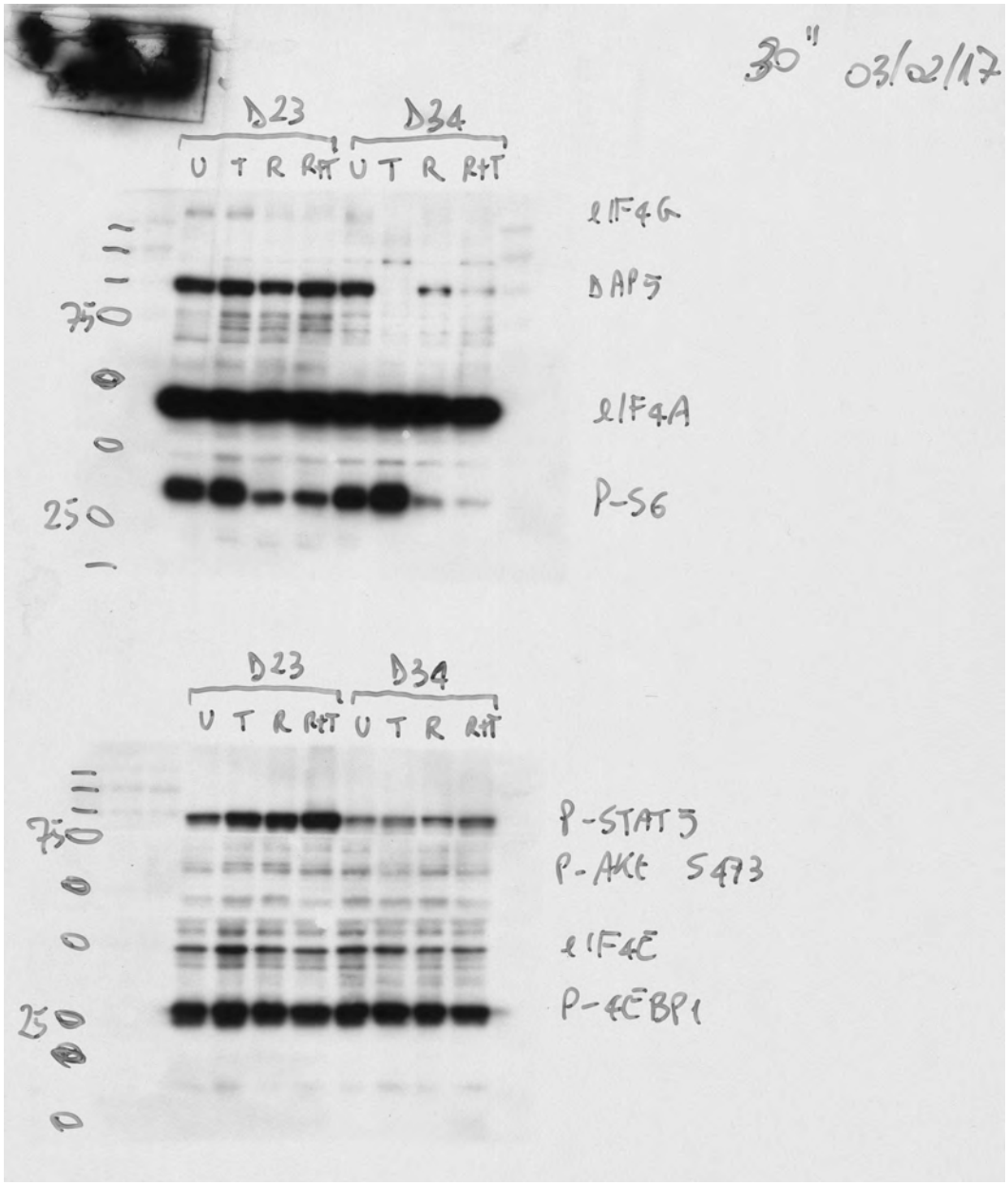

Supplement: Supplementary file 13 — Source Data [file 41467_2021_27087_MOESM13_ESM.zip › Source Data/Uncut immunoblots pdf files/Figure 4e immunoblots/030217_30s copy.tif.pdf]

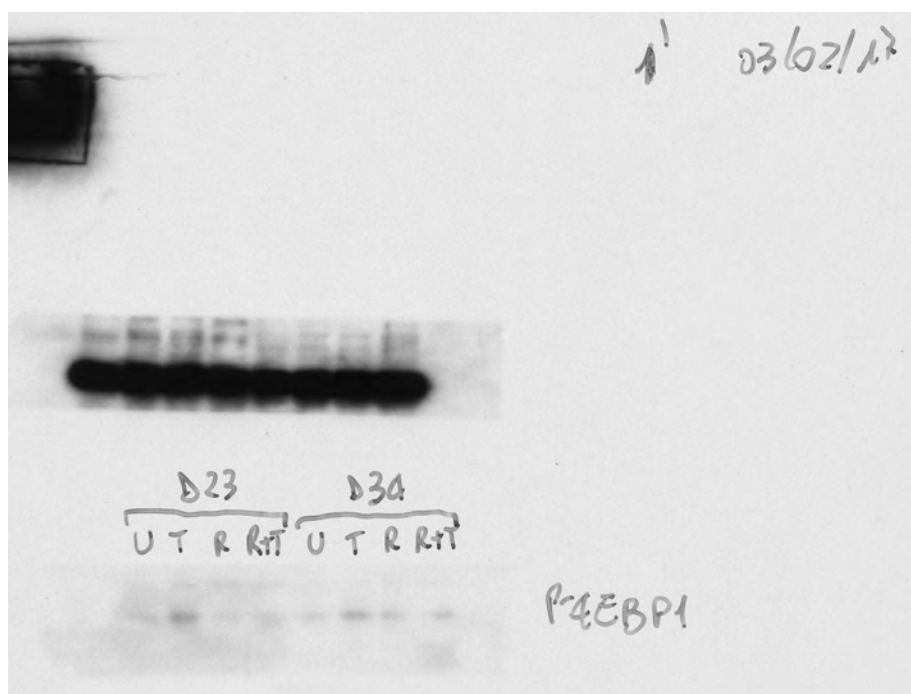

Supplement: Supplementary file 13 — Source Data [file 41467_2021_27087_MOESM13_ESM.zip › Source Data/Uncut immunoblots pdf files/Figure 4e immunoblots/030217_4A_4EBP1_1min copy.tif.pdf]

2' 03/02/17

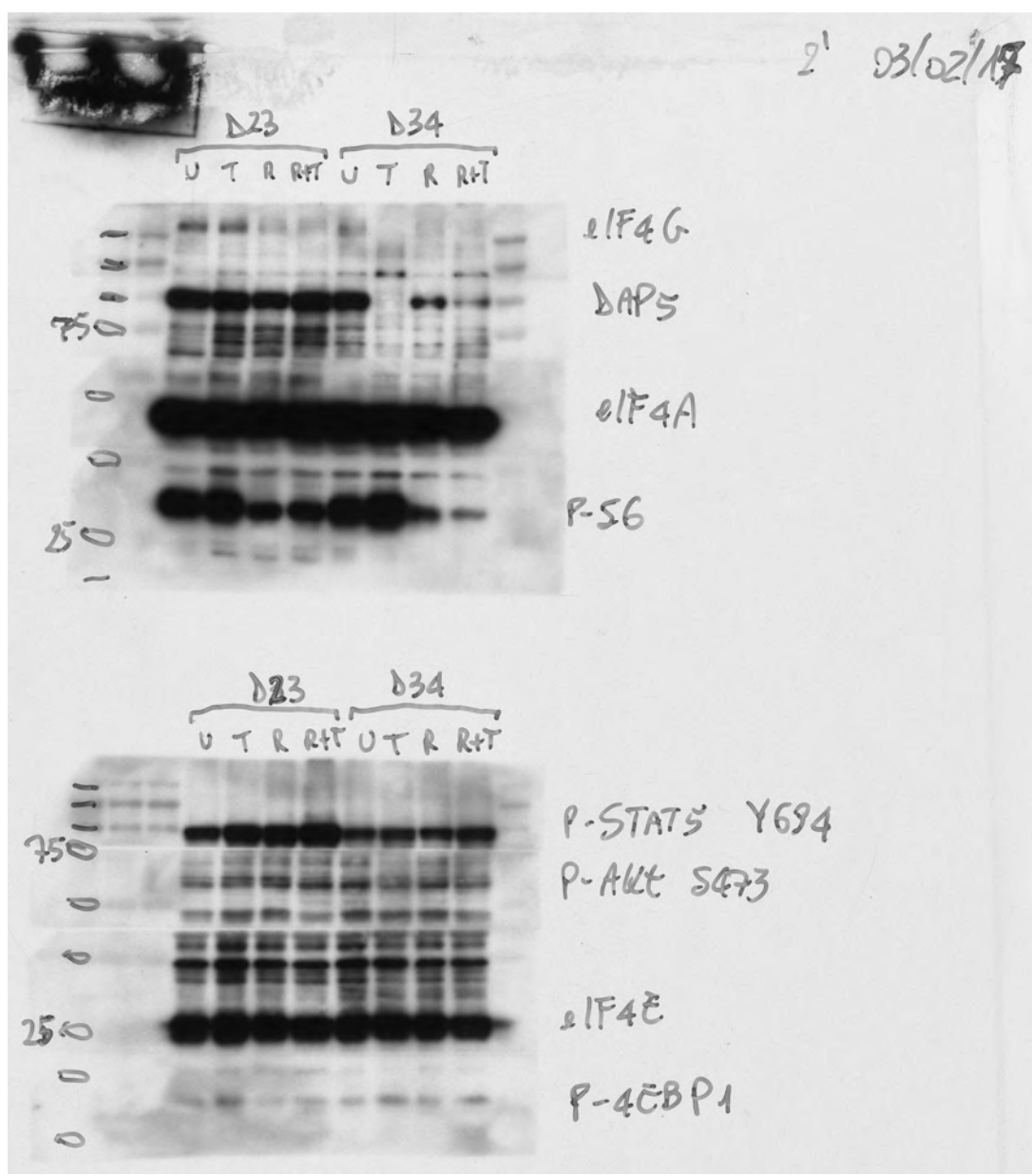

Supplement: Supplementary file 13 — Source Data [file 41467_2021_27087_MOESM13_ESM.zip › Source Data/Uncut immunoblots pdf files/Figure 4e immunoblots/030217_2min copy.tif.pdf]

1" 03/02/17

| D23 |   |   |    | D34 |   |   |    |
|-----|---|---|----|-----|---|---|----|
| U   | T | R | RT | U   | T | R | RT |

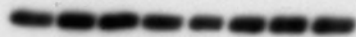

21F4A

Supplement: Supplementary file 13 — Source Data [file 41467_2021_27087_MOESM13_ESM.zip › Source Data/Uncut immunoblots pdf files/Figure 4e immunoblots/030217_4A_4EBP1_1s copy.tif.pdf]

1<sup>st</sup> 03/02/17

i

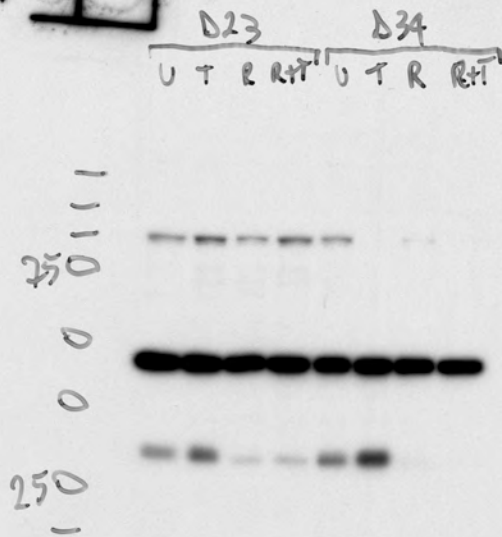

eIF4G

DAP5

eIF4A

p-S6

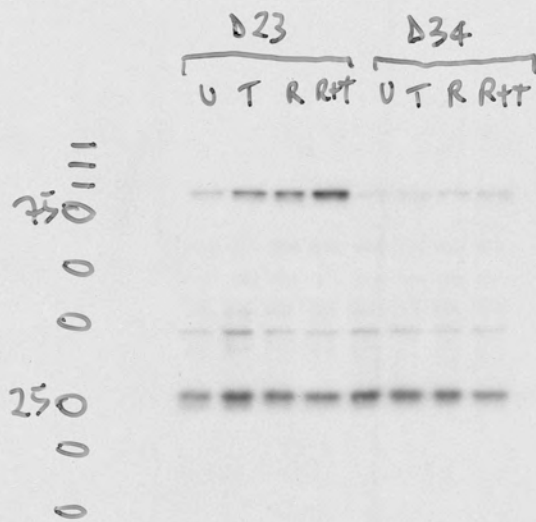

p-STAT5

p-AKT S473

eIF4E

Supplement: Supplementary file 13 — Source Data [file 41467_2021_27087_MOESM13_ESM.zip › Source Data/Uncut immunoblots pdf files/Figure 4e immunoblots/030217_1s copy.tif.pdf]

5" 03/02/17

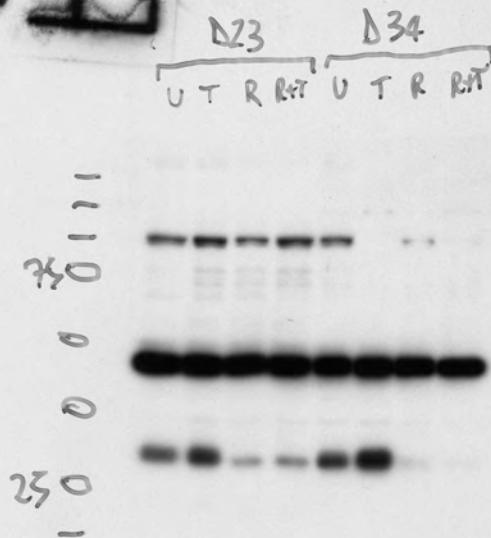

2IF4G

DAP5

2IF4A

P-S6

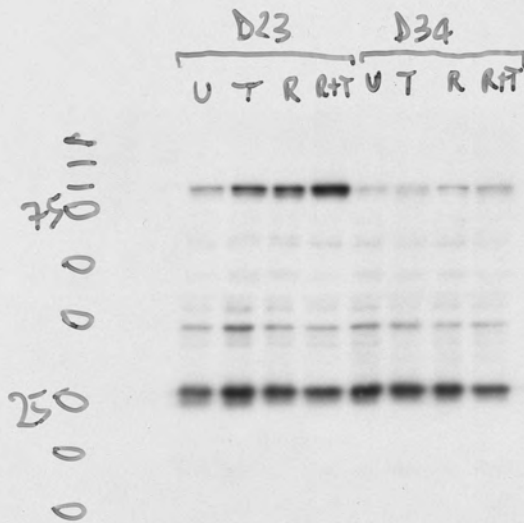

P-STAT5

P-AKT S473

2IF4E

Supplement: Supplementary file 13 — Source Data [file 41467_2021_27087_MOESM13_ESM.zip › Source Data/Uncut immunoblots pdf files/Figure 4e immunoblots/030217_5s copy.tif.pdf]

1' 03/07/17

D23 D34  
U T R R+T U T R R+T

1150  
0  
0  
250  
0  
0  
0

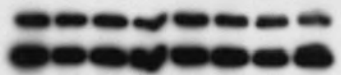

eIF4A  
GAPDH

MEMB 03/02/17

D23 D34  
U T R R+T U T R R+T

4EPR1 00

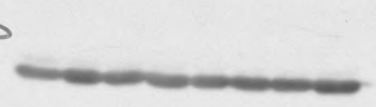

}  $\beta$ -actin

membrane 03/02/17

Supplement: Supplementary file 13 — Source Data [file 41467_2021_27087_MOESM13_ESM.zip › Source Data/Uncut immunoblots pdf files/Figure 4e immunoblots/030717_1min copy.tif.pdf]

5' 03/02/17

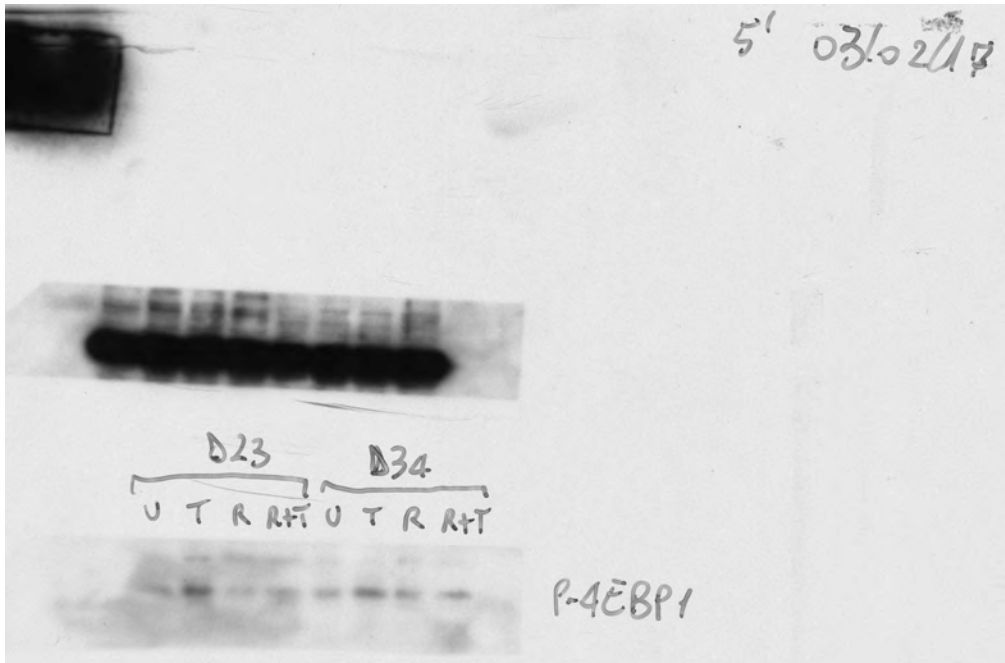

Supplement: Supplementary file 13 — Source Data [file 41467_2021_27087_MOESM13_ESM.zip › Source Data/Uncut immunoblots pdf files/Figure 4e immunoblots/030217_4A_4EBP1_5min copy.tif.pdf]

30" 03/03/17

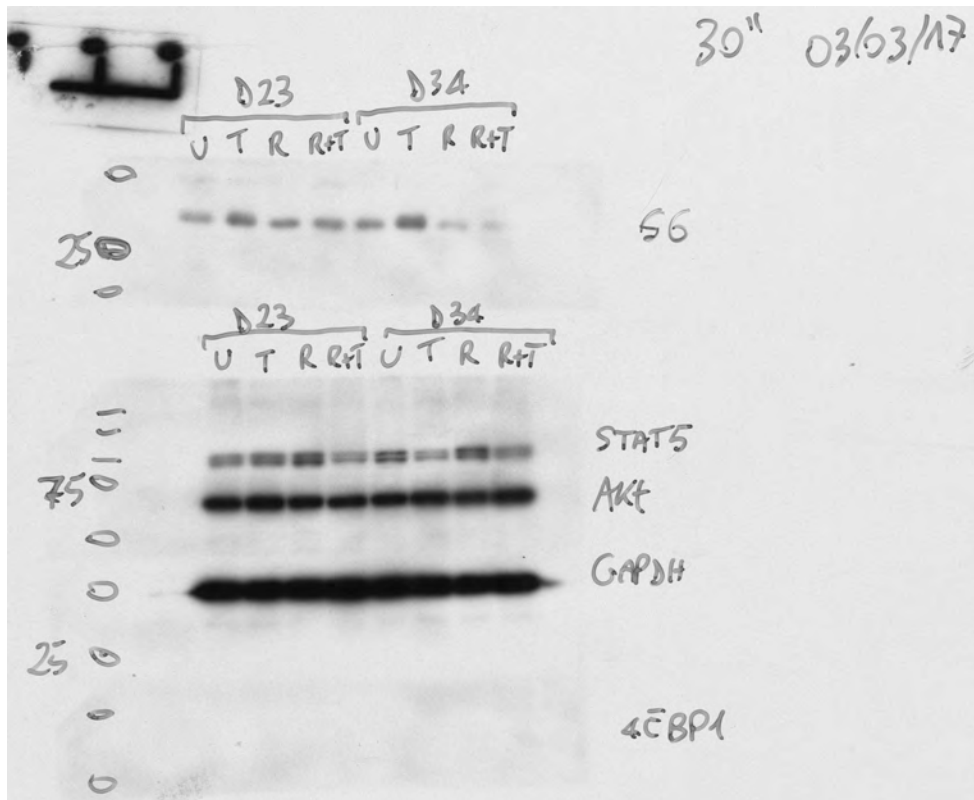

Supplement: Supplementary file 13 — Source Data [file 41467_2021_27087_MOESM13_ESM.zip › Source Data/Uncut immunoblots pdf files/Figure 4e immunoblots/030317_30s copy.tif.pdf]

5" 03/07/17

D23      D34  
U T R RT U T R RT

1100  
750  
500  
250  
200  
150  
100  
50

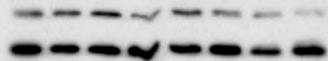

EIF44  
GAPDH

MEMB. 03/02/17

4EBP1 500

$\beta$ -actin

Supplement: Supplementary file 13 — Source Data [file 41467_2021_27087_MOESM13_ESM.zip › Source Data/Uncut immunoblots pdf files/Figure 4e immunoblots/030717_5s copy.tif.pdf]

10' 03/02/17

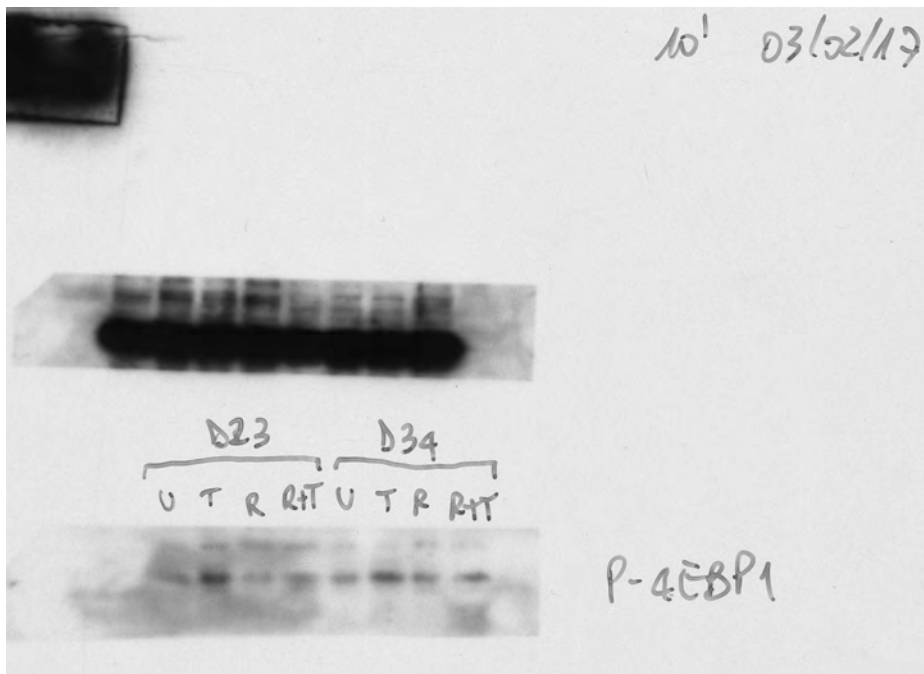

Supplement: Supplementary file 13 — Source Data [file 41467_2021_27087_MOESM13_ESM.zip › Source Data/Uncut immunoblots pdf files/Figure 4e immunoblots/030217_4A_4EBP1_10min copy.tif.pdf]

1' 03/03/17

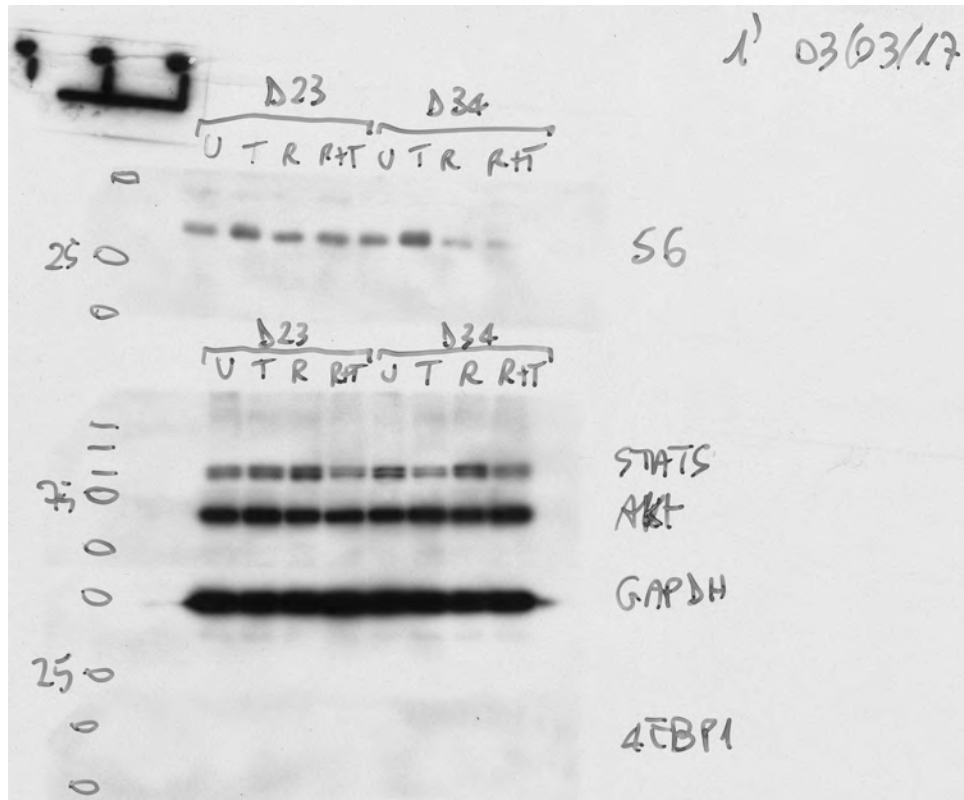

Supplement: Supplementary file 13 — Source Data [file 41467_2021_27087_MOESM13_ESM.zip › Source Data/Uncut immunoblots pdf files/Figure 4e immunoblots/030317_1min copy.tif.pdf]

1' 03/02/17

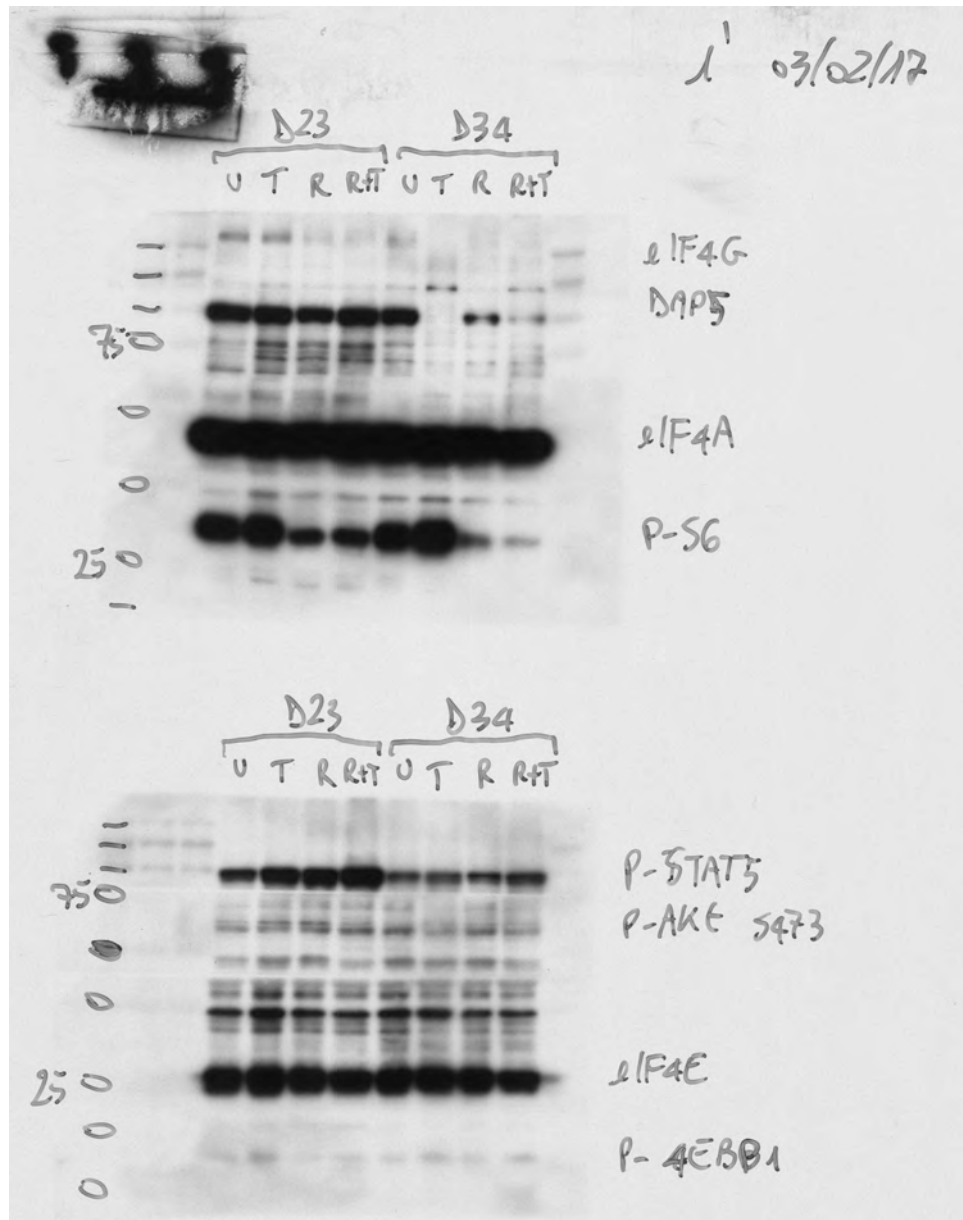

Supplement: Supplementary file 13 — Source Data [file 41467_2021_27087_MOESM13_ESM.zip › Source Data/Uncut immunoblots pdf files/Figure 4e immunoblots/030217_1min copy.tif.pdf]

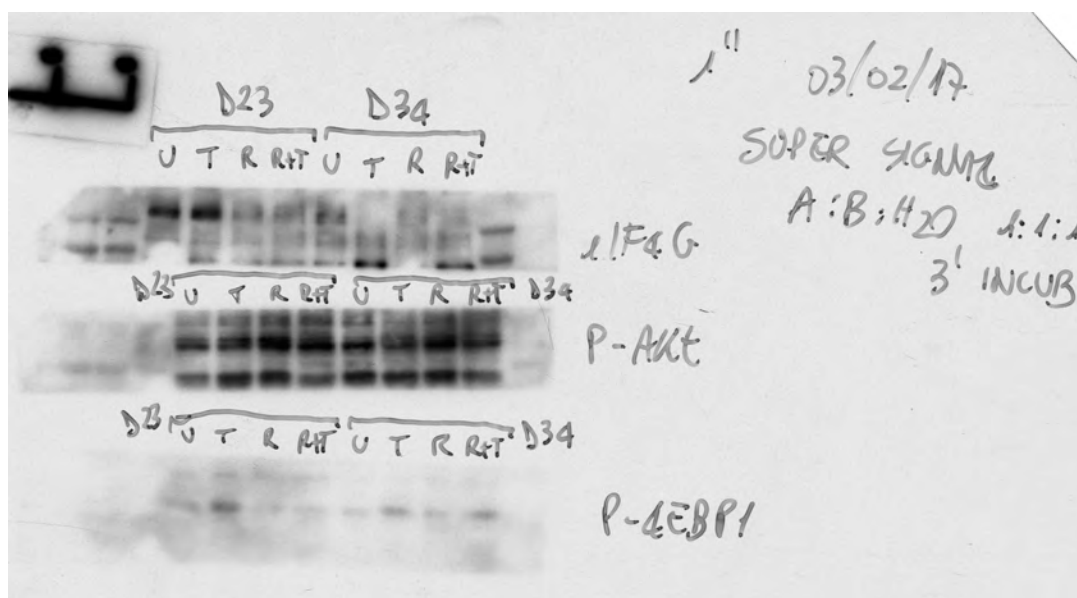

Supplement: Supplementary file 13 — Source Data [file 41467_2021_27087_MOESM13_ESM.zip › Source Data/Uncut immunoblots pdf files/Figure 4e immunoblots/030217_SS_1s copy.tif.pdf]

15" 03/07/17

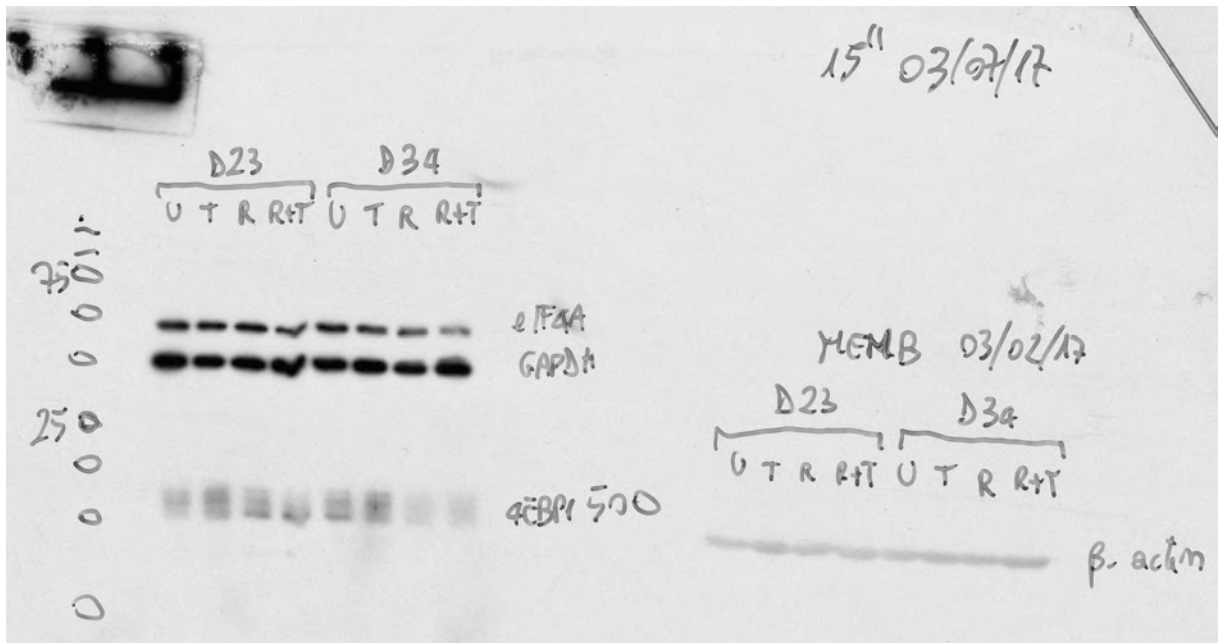

Supplement: Supplementary file 13 — Source Data [file 41467_2021_27087_MOESM13_ESM.zip › Source Data/Uncut immunoblots pdf files/Figure 4e immunoblots/030717_15s copy.tif.pdf]

5" 03/03/12

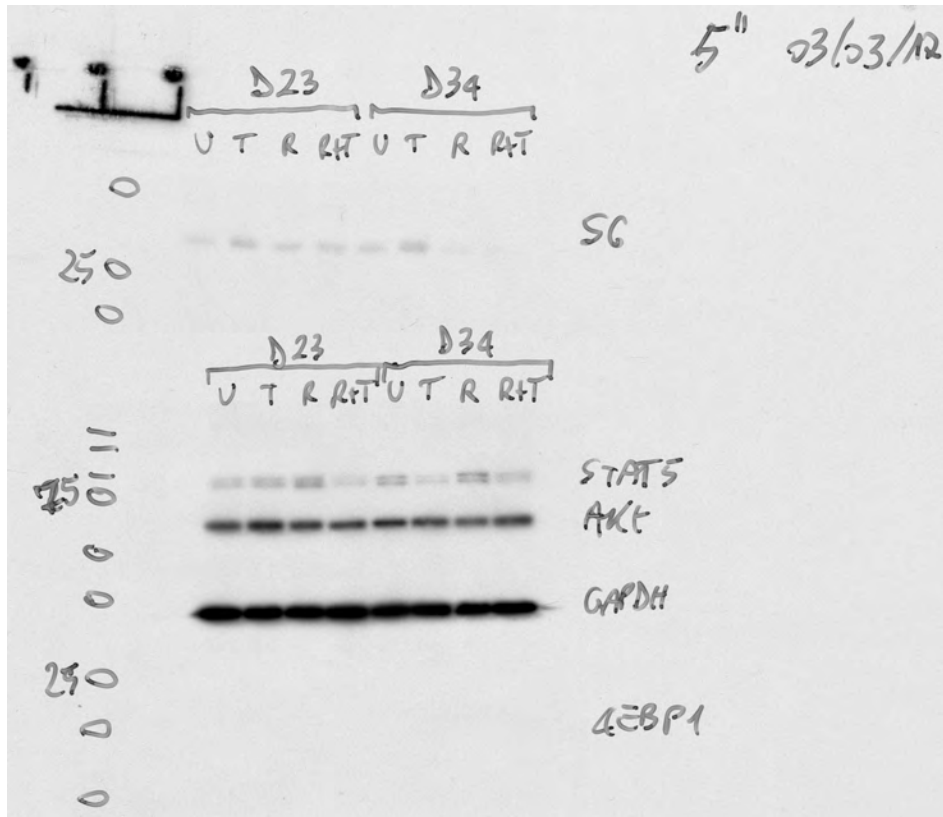

Supplement: Supplementary file 13 — Source Data [file 41467_2021_27087_MOESM13_ESM.zip › Source Data/Uncut immunoblots pdf files/Figure 4e immunoblots/030317_5s copy.tif.pdf]

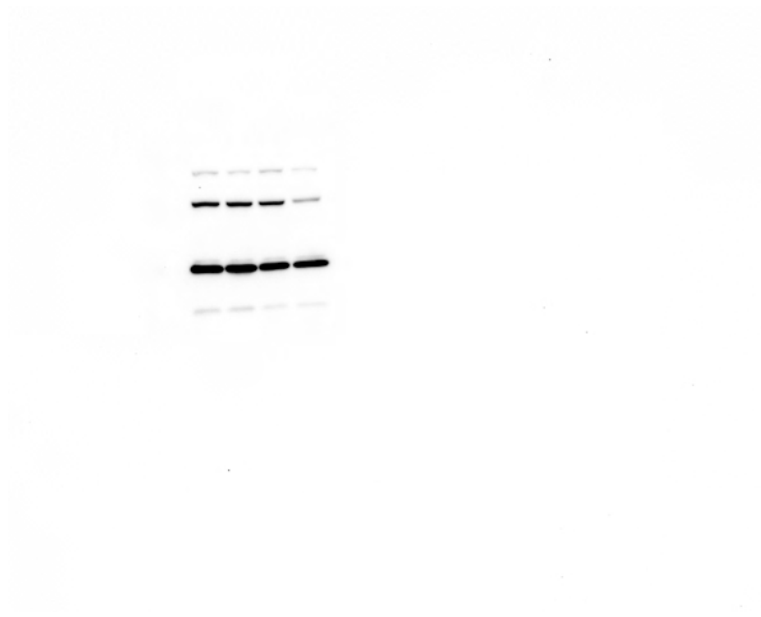

Supplement: Supplementary file 13 — Source Data [file 41467_2021_27087_MOESM13_ESM.zip › Source Data/Uncut immunoblots pdf files/Figure 7g immunoblots/2021_01_26_144434.tif.pdf]

Figure 7g  
Raw WB images

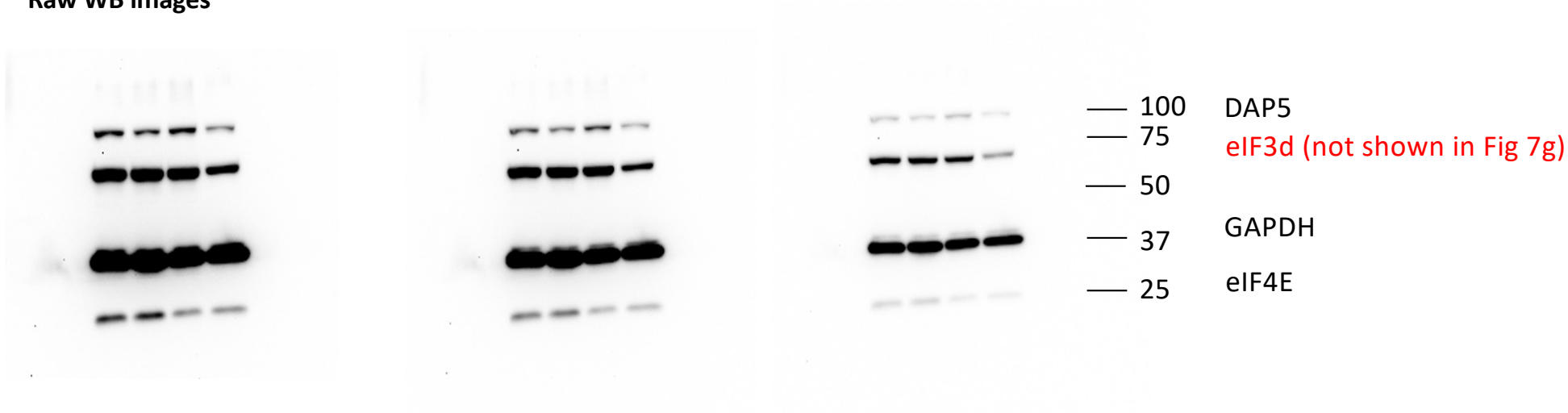

Supplement: Supplementary file 13 — Source Data [file 41467_2021_27087_MOESM13_ESM.zip › Source Data/Uncut immunoblots pdf files/Figure 7g immunoblots/Fig 7g.pdf]

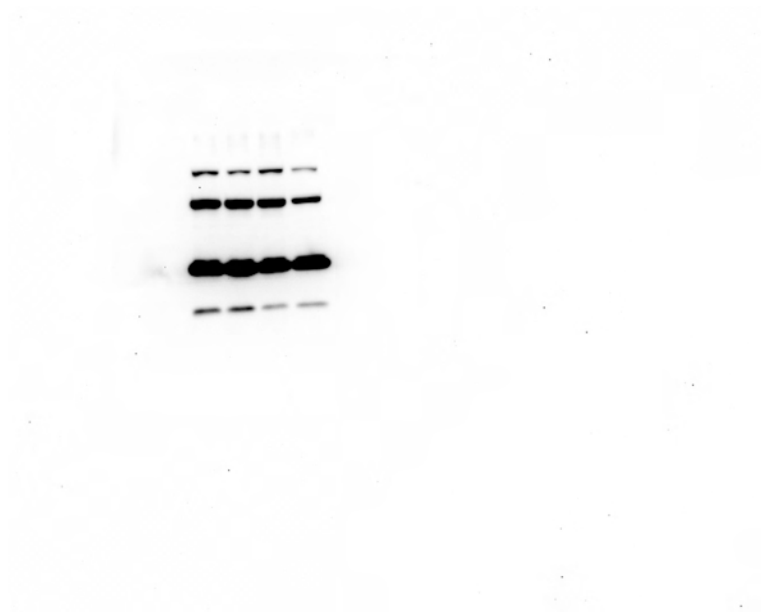

Supplement: Supplementary file 13 — Source Data [file 41467_2021_27087_MOESM13_ESM.zip › Source Data/Uncut immunoblots pdf files/Figure 7g immunoblots/2021_01_26_144616.tif.pdf]

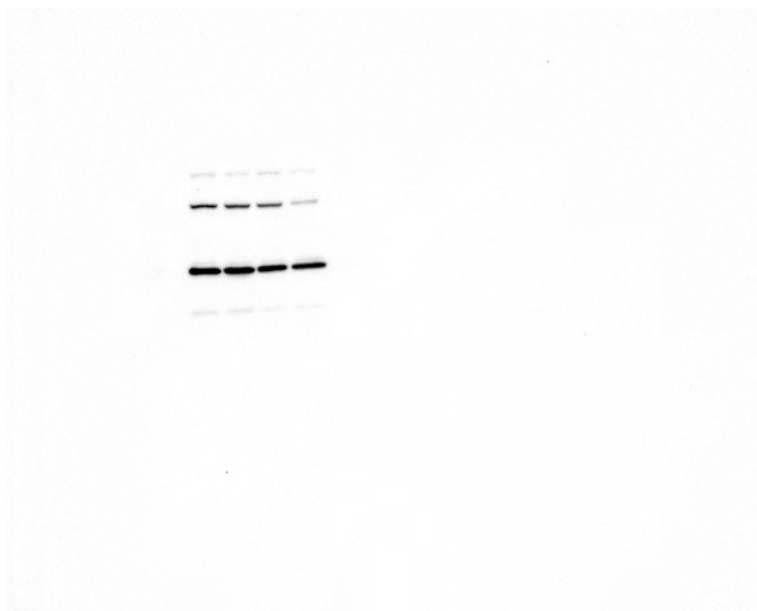

Supplement: Supplementary file 13 — Source Data [file 41467_2021_27087_MOESM13_ESM.zip › Source Data/Uncut immunoblots pdf files/Figure 7g immunoblots/2021_01_26_144538.tif.pdf]

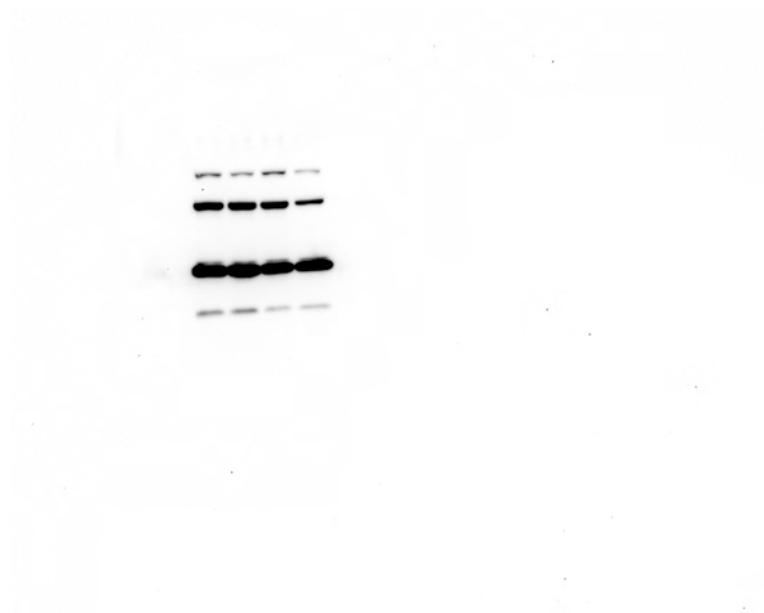

Supplement: Supplementary file 13 — Source Data [file 41467_2021_27087_MOESM13_ESM.zip › Source Data/Uncut immunoblots pdf files/Figure 7g immunoblots/2021_01_26_144504.tif.pdf]

**Figure 8C**

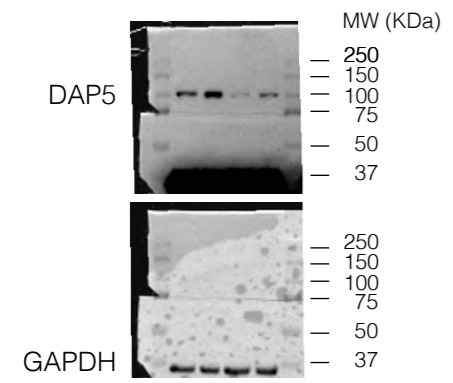

Supplement: Supplementary file 13 — Source Data [file 41467_2021_27087_MOESM13_ESM.zip › Source Data/Uncut immunoblots pdf files/Figure 8c immunoblots/Figure 8C raw WB.pdf]

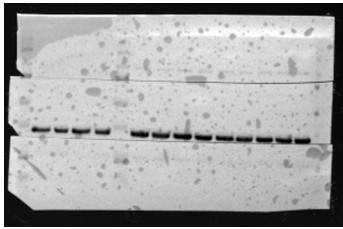

Supplement: Supplementary file 13 — Source Data [file 41467_2021_27087_MOESM13_ESM.zip › Source Data/Uncut immunoblots pdf files/Figure 8c immunoblots/2021_03_30_121735 membrane overlay 0.5s iTregs left.tif.pdf]

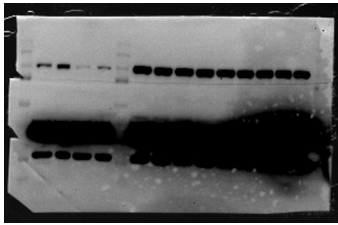

Supplement: Supplementary file 13 — Source Data [file 41467_2021_27087_MOESM13_ESM.zip › Source Data/Uncut immunoblots pdf files/Figure 8c immunoblots/2021_03_30_122048 membrane overlay 240s iTregs left.tif.pdf]

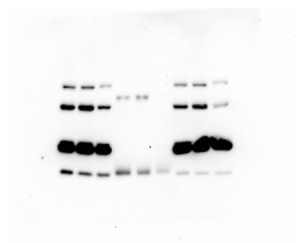

Supplement: Supplementary file 13 — Source Data [file 41467_2021_27087_MOESM13_ESM.zip › Source Data/Uncut immunoblots pdf files/Figure 7b immunoblots/2021_02_09_114852.tif.pdf]

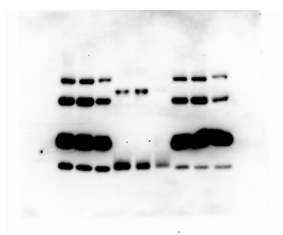

Supplement: Supplementary file 13 — Source Data [file 41467_2021_27087_MOESM13_ESM.zip › Source Data/Uncut immunoblots pdf files/Figure 7b immunoblots/2021_02_09_114938.tif.pdf]

Figure 7b  
Raw WB images

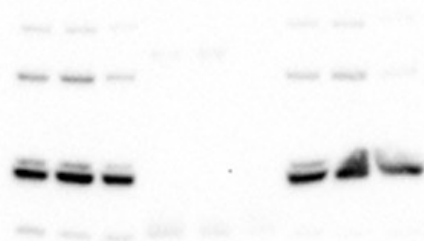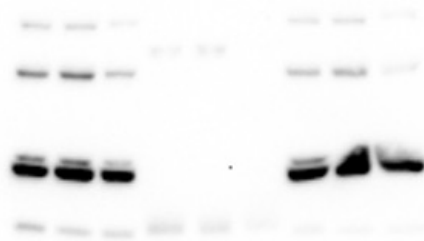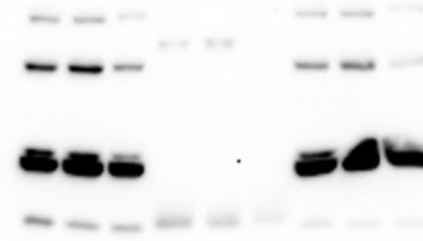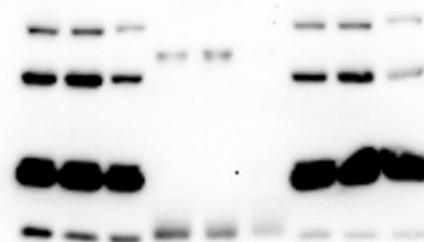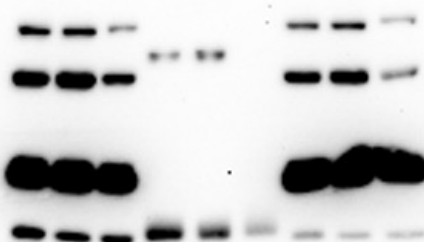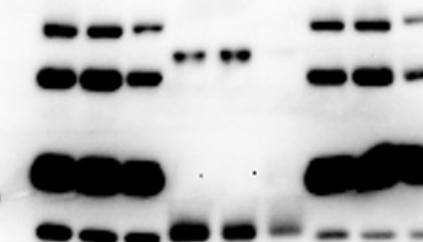

Supplement: Supplementary file 13 — Source Data [file 41467_2021_27087_MOESM13_ESM.zip › Source Data/Uncut immunoblots pdf files/Figure 7b immunoblots/Fig 7b.pdf]

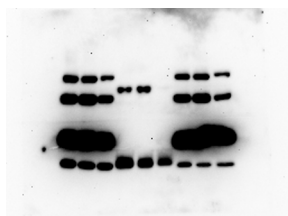

Supplement: Supplementary file 13 — Source Data [file 41467_2021_27087_MOESM13_ESM.zip › Source Data/Uncut immunoblots pdf files/Figure 7b immunoblots/2021_02_09_114044.tif.pdf]

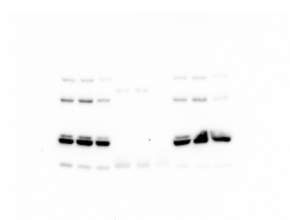

Supplement: Supplementary file 13 — Source Data [file 41467_2021_27087_MOESM13_ESM.zip › Source Data/Uncut immunoblots pdf files/Figure 7b immunoblots/2021_02_09_114805.tif.pdf]

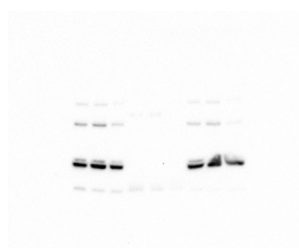

Supplement: Supplementary file 13 — Source Data [file 41467_2021_27087_MOESM13_ESM.zip › Source Data/Uncut immunoblots pdf files/Figure 7b immunoblots/2021_02_09_114728.tif.pdf]

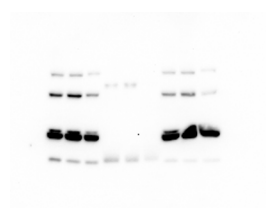

Supplement: Supplementary file 13 — Source Data [file 41467_2021_27087_MOESM13_ESM.zip › Source Data/Uncut immunoblots pdf files/Figure 7b immunoblots/2021_02_09_114826.tif.pdf]

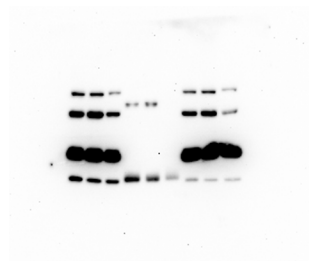

Supplement: Supplementary file 13 — Source Data [file 41467_2021_27087_MOESM13_ESM.zip › Source Data/Uncut immunoblots pdf files/Figure 7b immunoblots/2021_02_09_113909.tif.pdf]
